# Visible light-regulated cationic polymer coupled with photodynamic inactivation as an effective tool for pathogen and biofilm elimination

**DOI:** 10.1186/s12951-022-01702-4

**Published:** 2022-11-24

**Authors:** Qian Wang, Qingshan Shi, Yulian Li, Shunying Lu, Xiaobao Xie

**Affiliations:** Guangdong Provincial Key Laboratory of Microbial Culture Collection and Application, State Key Laboratory of Applied Microbiology Southern China, Institute of Microbiology, Guangdong Academy of Sciences, Guangzhou, 510070 People’s Republic of China

**Keywords:** Photodynamic inactivation, Antimicrobial polymers, Bacterial infections, Drug resistance, Pathogens, Biofilm

## Abstract

**Background:**

Pathogenic microorganism pollution has been a challenging public safety issue, attracting considerable scientific interest. A more problematic aspect of this phenomenon is that planktonic bacteria exacerbate biofilm formation. There is an overwhelming demand for developing ultra-efficient, anti-drug resistance, and biocompatibility alternatives to eliminate stubborn pathogenic strains and biofilms.

**Results:**

The present work aims to construct a visible light-induced anti-pathogen agents to ablate biofilms using the complementary merits of ROS and cationic polymers. The photosensitizer chlorin e6-loaded polyethyleneimine-based micelle (Ce6-TPP-PEI) was constructed by an amphiphilic dendritic polymer (TPP-PEI) and physically loaded with photosensitizer chlorin e6. Cationic polymers can promote the interaction between photosensitizer and Gram-negative bacteria, resulting in enhanced targeting of PS and lethality of photodynamic therapy, and remain active for a longer duration to prevent bacterial re-growth when the light is turned off. As expected, an eminent antibacterial effect was observed on the Gram-negative *Escherichia coli*, which is usually insensitive to photosensitizers. Surprisingly, the cationic polymer and photodynamic combination also exert significant inhibitory and ablative effects on fungi and biofilms. Subsequently, cell hemolysis assessments suggested its good biocompatibility.

**Conclusions:**

Given the above results, the platform developed in this work is an efficient and safe tool for public healthcare and environmental remediation.

**Supplementary Information:**

The online version contains supplementary material available at 10.1186/s12951-022-01702-4.

## Introduction

Pathogenic microorganism pollution has been an escalating global health threat to human society, which would cause serious infection and even lead to death [1]. Furthermore, antibiotic-resistant pathogens (ARPs) worsen the problem due to the widespread and indiscriminate use of antibiotics [2, 3]. More worrisome is that planktonic pathogens tend to adhere to the surface of the substance and subsequently colonize to a sessile community through a quorum sensing system to form biofilms. These biofilms are complex, multicellular bacterial communities embedded in self-secreted, extracellular polymeric substances (EPS) [4, 5]. The formed biofilms block the penetration of small molecule antimicrobial agents, ultimately leading to insensitivity to antimicrobial agents, with up to 1000-fold greater effective doses compared to planktonic cells [6–8]. Therefore, the matrix provides physical protection, resulting in biofilm infections that are notoriously difficult to treat [9].

Over the past decades, unremitting efforts have been made by scientific communities and pharmaceutical industries to tackle this worldwide dilemma. Notably, nanomaterials have exhibited remarkable success in antibacterial applications [10–12]. For example, nanomaterials serve as delivery vehicles or facilitate the targeted bacterial permeability to couple with bacteriophages [13], antimicrobial peptides [14, 15], cationic compounds [16, 17], photodynamic therapy [18, 19] and photothermal therapy (PTT) [20, 21]. They have been widely used in various fields, including water disinfection, the marine industry, biomedicine, the textile industry and food packaging, antibacterial coatings, for biofilm elimination, and combating antibiotic-resistant pathogens. For example, a structure-changed nanomaterial (CS-b-PEG) fabricated by a block chitosan and poly(ethylene glycol) copolymer, which is without biocides releasing behavior was designed to in marine fields [22].

Due to its good bactericidal efficiency, the new promising light-activated alternative, photodynamic antimicrobial chemotherapy (PACT), characterized by broad-spectrum, non-invasiveness, and controllability, has attracted considerable attention [23]. Its mechanism involves utilizing of the photochemical reactions of non-toxic photosensitizers (PSs). Upon irradiation with light whose wavelength matches the absorption of the PSs, the generation of toxic reactive oxygen species (ROS), could cause irreparable damage to the cell membrane and intracellular DNA [24] [25]. However, some drawbacks diminish PACT efficiency, such as poor water solubility, the short lifetime and limited diffusion distance of ROS, and weak interaction between the PSs and Gram-negative bacteria [24, 26, 27]. With advances in nanotechnology, researchers have developed a variety of delivery vehicles, such as polymers, metal nanoparticles, and metal–organic frameworks (MOFs) [28–31], to further improve the interactions between PSs and pathogens, i.e., targeting ability and high adherence, to enhance PACT efficiency.

Meanwhile, another hallmark of PACT is that the ROS generation may cease after the light irradiation is turned off, allowing un-killed bacteria to proliferate [32]. It is still uncertain whether pathogens are capable of developing resistance to ROS through antioxidant enzyme activation or other possible mechanisms [33, 34]. To avoid the emergence of PACT drug-resistant strains, it is not sufficient for delivery vehicles to only deliver photosensitizers.

A balanced hydrophilic-hydrophobic ratio of polymer would enable the synthesized product to self-assemble and form micellar aggregates in water as a delivery vehicle to construct nano-photosensitizers for enhanced photodynamic therapy. More delightfully, It was reported that positively charged nanocarriers could efficiently penetrate biofilms [35]. These positively charged nanoparticles not only exhibit long-term antibacterial effects after PACT but also provide a solution to remove stubborn bacterial biofilms. However, the ability to inhibit and remove biofilms has not been fully explored.

Consequently, we reported the construction of photosensitizer chlorin e6-loaded polymeric nanoparticles (Ce6-TPP-PEI) via the self-assembly of amphiphilic antibacterial polymers (TPP-PEI). With the combination of photodynamic therapy and cationic antimicrobial polymers, the nanoparticles exhibited an inactivation rate of about 99.99% on *Escherichia coli*, which is usually insensitive to photodynamic therapy, with visible light irradiation for 5 min. Along with high efficiency of the anti-pathogenic agent, excellent biofilm removal efficacy was observed, providing a promising prospect in disinfection. Amphiphilic cationic polymer, prepared from 4-carboxbutyltriphenylphosphonium bromide and branched polyethyleneimine (10 kD), can provide an anti-pathogenic microorganism complementary therapy at the end of PACT, further offer long-lasting bacterial inhibition effects and preventing secondary infection caused by incompletely killed bacteria.

Fungal infections currently affect nearly a quarter of the worldwide population [36]. However, significantly fewer fungicides have been explored and introduced to clinical practice compared with antibacterial drugs until now [37]. Unfortunately, the drug resistance of pathological fungi is constantly growing. We also confirm that Ce6-TPP-PEI could inactivate the pathogenic fungi. *Candida albicans*, which has accounted for over half the reported cases of invasive candidiasis, served as a model to investigate the inactivation properties of Ce6-TPP-PEI [38]. Taken together, these photosensitizer Ce6-loaded antibacterial polymeric nanoparticles effectively inactivate pathogenic microorganism, including but not limited to bacteria and *C. albicans*, but also notorious biofilms, showing great potential for new strategies and more effective PACT-based agents in the future.

## Materials and methods

### Materials

N, N-dimethyl formamide (DMF), ether, benzene, hexane and acetonitrile were obtained from Guangzhou Chemical Reagent Factory (Guangzhou, China). Branched polyethyleneimine (PEI, 10 kD) was provided by Innochem (Beijing) Technology Co., Ltd. Dicyclohexylcarbodiimide (DCC) and 4-(dimethylamino) pyridine (DMAP) were purchased from Alfa Aesar. PS Chlorin e6 (Ce6), 5-bromovaleric acid (Br-C_4_H_8_-COOH) and triphenylphosphine (TPP) were obtained from Macklin. Dichlorofluorescein diacetate (DCFH-DA) was purchased from Sigma Aldrich Chemicals (St. Louis, MO, USA). SYTO (KFS147) was provided by Beijing Baiao Laibo Technology Co. Ltd. Propidium iodide (PI) was acquired from Invitrogen. Luria–Bertani (LB) agar was acquired from Guangdong Huankai Microbial Sci. and Tech. Co., Ltd. (Guangzhou, China). All the solvents and reagents were of analytical grade unless specified otherwise. Secondary reverse osmosis water was made in our laboratory using a water purifier.

*Escherichia coli* (*E. coli*, ATCC 25922), *Bacillus subtilis* (*B. subtilis*, ATCC 6633) and *Candida albicans* (*C. albicans*, ATCC 10231) were supplied by the Guangdong Institute of Microbiology (Guangzhou, China) and cultured in Luria–Bertani (LB) medium, potato dextrose broth (PDB) or M9 minimal medium consisting of 1-g/L NH_4_Cl, 11-g/L Na_2_HPO_4_·7H_2_O, 3-g/L KH_2_PO_4_, 5-g/L NaCl, 4-g/L glucose,120-mg/L MgSO_4_, and 10-mg/L CaC1_2_ [39]. Bacterial cells were cultured using LB liquid and solid media under an aerobic atmosphere.

### Synthesis

#### Preparation of 4-carboxbutyltriphenylphosphonium bromide

5-bromovaleric acid (1 g, 5.5 mmol) was pre-dissolved in 5 mL of acetonitrile, then triphenylphosphine (1.6 g, 6.1 mmol) was added to the above solution and refluxed at 80 °C for 24 h. After the reaction, the solvent was dried with a rotary evaporator, and the remaining solid was re-dissolved in dichloromethane. The above dichloromethane solution was precipitated in cold ether to obtain a white solid. Subsequently, it was filtered, collected, and rinsed with benzene, n-hexane and ether in turn, and then dried in vacuum to obtain the final product.

#### Preparation of TPP-PEI

4-Carboxbutyltriphenylphosphonium bromide and DMAP were pre-dissolved in 20 mL of anhydrous dichloromethane and stirred for 1 h in an argon atmosphere. DCC and branched polyethyleneimine were dissolved in 3 mL of anhydrous DMF and dropped into the above solution. After reacting for 24 h at 0 °C, the white precipitate was removed by filtration. Finally, the obtained solution was transferred into a dialysis tube with molecular weight of 3500 and dialyzed with ultrapure water for 48 h. During dialysis, ultrapure water was changed every 6 h. After dialysis, the aqueous solution was lyophilized and stored for further use.

#### Preparation of TPP-PEI and Ce6-TPP-PEI

TPP-PEI (20 mg) was pre-dissolved in DMF (5 mL), then dropwise added to 20 mL deionized water. After vigorously stirring for 2 h, the above solution was transferred to a dialysis tube (MWCO 3500). With the water being changed at fixed time intervals, the solution was dialyzed against ultrapure water for 48 h at 25 °C to obtain the final product. Similar method as above was used to obtain the Ce6-loaded micelles (Ce6-TPP-PEI). Photosensitizer Ce6 (2 mg) and TPP-PEI (20 mg) were dissolved by DMF (5 mL) in advanced and stirred for 2 h. Subsequently, the obtained solution was dialyzed against ultrapure water at room temperature for 48 h with a dialysis tube (MWCO 3500 Da). Finally, the final suspension was filtered through a syringe filter (a pore size of 450 nm) and stored at 4 °C before further experiments usage.

### Characterizations

Transmission electron microscopy (TEM, 80 kV, Hitachi, H-7650) was used to record the size and morphology of Ce6-TPP-PEI in the aqueous solution. Zeta-sizer instrument (Zetasizer Pro, Malvern Instruments, UK) was served to investigate the average hydrodynamic particle size and polydispersity index. The UV/Vis absorption spectra of free Ce6, TPP-PEI and Ce6-TPP-PEI were recorded by Lambda 45 UV/Vis Spectrometer (Perkin-Elmer, USA). Fourier transform infrared spectra (FT-IR) were investigated on an FT-IR spectrophotometer (VERTEX 70, Bruck, Germany) using the KBr pellet technique.

The Ce6 loading capacity (LC) and encapsulation efficiency (EE) in polymeric Ce6-TPP-PEI micelles were measured according to our previous study [24]. The concentrations of Ce6 were calculated by the standard curve, based on known concentrations of Ce6 in DMSO and UV/Vis absorbance. Firstly, the standard curve of the known concentration of Ce6 (in DMSO) and the absorbance at 405 nm was recorded by Lambda 45 UV/Vis Spectrometer. Then, the solution of Ce6-TPP-PEI (1 mL) was freeze-dried, weighed and re-dissolved in DMSO, and the UV/Vis absorbance was further measured at 405 nm. The EE and LC of Ce6 were calculated separately according to the following formula:

$${\text{LC }}\left( \% \right) = \left( {{\raise0.7ex\hbox{${\text{amount of loaded Ce6}}$} \!\mathord{\left/ {\vphantom {{\text{amount of loaded Ce6}} {{\text{amount of Ce6}} - {\text{loaded nanocarrier}}}}}\right.\kern-\nulldelimiterspace} \!\lower0.7ex\hbox{${{\text{amount of Ce6}} - {\text{loaded nanocarrier}}}$}}} \right) \times 100$$

$${\text{EE }}\left( \% \right) = \left( {{\raise0.7ex\hbox{${\text{amount of loaded Ce6}}$} \!\mathord{\left/ {\vphantom {{\text{amount of loaded Ce6}} {\text{initial amount of Ce6}}}}\right.\kern-\nulldelimiterspace} \!\lower0.7ex\hbox{${\text{initial amount of Ce6}}$}}} \right) \times 100$$

### The detection of reactive oxygen species

DCFH was served as a fluorescent labeled probe to detect the ROS produced of Ce6-TPP-PEI. Firstly, DCFH-DA was hydrolyzed to DCFH according to our previous literature [24]. DCFH (100 µL, 5 µM) solution was mixed with Ce6-TPP-PEI (100 µL, 2.5 µM) in a 96 well plate at room temperature, while a solution of TPP-PEI and PBS mixed with DCFH solution was used as a control. Then, the mixture was irradiated or not for different times with a commercially available LED light (λmax = 630 nm, 30 mW/cm^2^) with full width at half maximum of 20 nm. The excitation filter was fixed at 488 nm, and the emission wavelength of the solution was recorded at 525 nm using a microplate reader (Synergy H1, BioTek, USA). Each sample was repeated three times for reproducibility.

### Confocal laser scanning microscopy observation of cellular uptake

Confocal laser scanning microscopy (CLSM) imaging was used to record the localization of Ce6-TPP-PEI in *E. coli* cells. Briefly, a freezer stock of *E.coli* was inoculated into LB liquid medium in advance and cultured to logarithmic phase at 37 °C after 12 h under shaking at 180 rpm. After centrifugation at 5000 rpm for 5 min, the bacterial cells were washed with normal saline solution (0.9% NaCl) three times. 500 µL of resuspended bacterial cells (10^8^ CFU/mL) were incubated with Ce6-TPP-PEI for 30 min. Finally, the bacterial solution was centrifuged and re-suspended in PBS for further observation with CLSM (LSM 800, Zeiss).

### Antibacterial Kinetics

Optical density at 600 nm (OD_600_) was served to determine the growth state of the bacteria. The typical Gram-negative bacteria *E. coli* and Gram-positive bacteria *B. subtilis* were chosen as models and were cultured as described above. Simply, *E.coli* or *B. subtilis* was inoculated into LB liquid medium and cultured to logarithmic phase at 37 °C under shaking at 180 rpm. Fresh *E. coli* or *B. subtilis* (100 µL, M9 medium) cells, pre-treated by centrifugation, washed, and resuspended, were inoculated into a 96-well plate at 10^5^ cells per well. Subsequently, another 100 µL of the medium containing different particles at various concentrations of Ce6 was added to the wells. After 30 min, the plates were irradiated with LED light (630 nm, 30 mW/cm^2^, 3 min), and cultured at 37 °C for 24 h. A microplate reader (TECAN, SPARK) was used to record the absorbance of the solution at 600 nm every hour. Each sample was repeated three times for reproducibility.

### Comparison of antibacterial activity

The colony forming method was adopted to further compare the antimicrobial activities. *E.coli* or *B. subtilis* were cultured and pre-treated as described above in “Confocal laser scanning microscopy observation of cellular uptake”. Firstly, the re-suspended bacterial cells (10^7^ CFU/mL) were incubated with Ce6-TPP-PEI, TPP-PEI, or free Ce6 for 30 min, with or without LED light irradiation (630 nm, 30 mW/cm^2^, 5 min), and diluted by 10^2^, 10^4^, and 10^6^ times. Finally, the treated bacterial suspension (100 µL) was separately transferred to LB agar medium, and cultured at 37 °C for 48 h to count the colony. The cells without nanomaterials were set as the control group. Concerning the comparison of antifungal ability, *C. albicans* was first cultured in a PDB liquid medium overnight and harvested. Subsequently, the cells (10^5^ CFU/mL) were incubated with Ce6-TPP-PEI, TPP-PEI, or free Ce6 for 30 min, with or without LED light irradiation (630 nm, 30 mW/cm^2^, 3 min) and diluted by 10^1^, 10^2^, and 10^4^ times. The treated bacterial suspension (100 µL) was separately transferred to Sabouraud agar medium, and cultured at 37 °C for 48 h to count the colony.

### Live/Dead bacterial viability assay

The antibacterial activity of Ce6-TPP-PEI and TPP-PEI against *E. coli* was visualized by using fluorescent nucleic acid-labeled probe SYTO 9 and PI. *E. coli* cells were cultured and pretreated as described in the antibacterial kinetics section. The resuspended bacteria (10^8^ CFU/mL) were incubated with materials solution in a centrifuge tube for 30 min, and then illuminated under LED light irradiation (630 nm, 30 mW/cm^2^, 3 min) or without irradiation. The bacterial cells were harvested by centrifugation and washed with 0.9% NaCl. The bacteria pellet was resuspended in sterilized water, followed by adding a mixture (2 μL) of the SYTO 9 and PI dyes to the solution, and incubated at room temperature for 20 min in the dark. The imaging of the fluorescence signal was recorded by Gen5 Imaging (BioTek, USA). The microbial cells treated with PBS were set as a control. Subsequently, the signal intensity was measured by a microplate reader (Synergy H1, BioTek, USA). The excitation filter was fixed at 488 nm, and the emission wavelength was recorded of 510–690 nm for each sample. The Red/Green ratio of the integrated intensity was calculated, which can indirectly reflect the ratio of live/dead to the sample. The integration intensity of green fluorescence ranged from 510 to 540 nm, while red fluorescence ranged from 620 to 660 nm.

### Inhibition of bacterial biofilm formation

The classical crystal violet (CV) staining method was used to determine the capability of Ce6-TPP-PEI to inhibit the formation of *E. coli* biofilms. Briefly, *E.coli* was inoculated into LB liquid medium and cultured to logarithmic phase at 37 °C under shaking at 180 rpm. Fresh *E. coli* cells (200 µL, M9 medium), pre-treated with centrifugation, were washed, re-suspended, and inoculated into a 48-well polystyrene plate at 10^8^ cells per well. Subsequently, another 200 µL of M9 medium containing various nanoparticles was added into the wells to culture for 30 min. The plate was then irradiated with LED light (630 nm, 30 mW/cm^2^, 5 min) and cultured at 37 °C for another 48 h. Subsequently, the medium was discarded, and the formed biofilm was washed twice with PBS to remove planktonic bacteria. Then 600 μL of CV (0.1% (w/v) in 0.9% NaCl) was added to each well and incubated at 37 °C for 30 min in the dark. The plate was washed for twice to remove the residual crystal violet, followed by imaging for the formed biofilms. To quantitatively analyze the formed biofilm, 600 μL of 95% ethanol was added to each well and incubated at 37 °C for 15 min at RT. The optical density was recorded at 590 nm using a microplate reader (BioTek, Synergy H1). Each sample was repeated three times for reproducibility.

### Ablation effect of bacterial preformed biofilms

The CV staining assay was used to quantitatively analyze the ability of Ce6-TPP-PEI to ablate the preformed *E. coli* biofilms. Firstly, *E.coli* was cultured overnight to the logarithmic phase, harvested by centrifugation, washed and resuspended. Then fresh *E. coli* cells (400 µL, 10^8^, M9 medium) were seeded into a 48-well polystyrene plate. Following 48 h of incubation to form the biofilms, the plate was washed with PBS three times to remove the planktonic bacteria. 400 μL of M9 medium with Ce6-TPP-PEI was added to the 48 well plates and cultured at 37 °C for 30 min, the plate was then irradiated with 630 nm LED light at a light intensity of 30 mW/cm^2^ for 5 min. After another 24 h of incubation, the medium was discarded, and the residual biofilms were washed with 0.9% NaCl for twice. 600 μL of CV (0.1% (w/v) in 0.9% NaCl) was added to each well and incubated at 37 °C for 30 min in the dark. Then CV solution was discarded, and the plate was washed with PBS twice to remove the residual crystal violet, followed by imaging for the formed biofilms. Concerning the quantitative analysis of the formed biofilm, 600 μL of 95% ethanol was used to dissolve the residual crystal violet and the optical density was recorded at 590 nm using a microplate reader (BioTek, Synergy H1). Data are presented as average ± SD (n = 3).

### Antifungal activity

The typical opportunistic pathogen *C. albicans* was chosen as a model to investigate the antifungal activity. *C. albicans* was first cultured in the PDB liquid medium and grew to the logarithmic phase at 37 °C under shaking at 180 rpm. Subsequently, the cells were centrifugated, washed with 0.9% NaCl twice, and harvested, followed by diluting into fresh PDB media to achieve a concentration of 10^5^ cells/mL. 100 µL aliquot of cells solution in sterilized water was seeded into a 96-well plate. Another 100 μL of serial dilutions material solution in H_2_O was separately added to the wells and incubated at 37 °C for 30 min. Then, the cells were irradiated with a 630-nm (30 mW/cm^2^, 5 min) LED light. The plate was centrifuged, and the supernatant was removed; 200 μL of fresh PDB media was added to each well. The suspension was mixed thoroughly and continued to be cultured for 48 h. The optical density was determined at 600 nm by spectrophotometry using a microplate reader (TECAN, SPARK) at fixed time intervals. Microbial cells incubated in the absence of material solution were set as a control. Each sample was repeated three times for reproducibility. The relative OD_600_ was calculated as A_600_/A_0600_, where A_0600_ is the turbidity at 600 nm of the freshly prepared bacteria solution, and A_0600_ is the turbidity at 600 nm of the *C. albicans* treated with sample and irradiation.

### The morphologie analysis of bacterial damage

Microscope cover slips were first immersed in 75% (v/v) ethanol for 24 h and washed with 0.9% NaCl three times to remove surface residues. A 48-well plate was pre-seeded with *E. coli* suspension (10^8^ CFU/mL, 1 mL), then the microscope cover slips were placed in the wells and incubated at 37 °C for 6 h under a static conditions. Subsequently, the medium was discarded, and the slips were washed with PBS three times. Ce6-TPP-PEI (4 μg/mL, 1 mL) was added to the wells to incubate for another 30 min, followed by rinsing with PBS three times to keep the cells free of polymers. The bacteria were first fixed with 3% glutaraldehyde for 5 h, exposed to an ethanol dehydration series of 30, 50, 70, 90, and 100% (v/v), and finally treated by tert-butanol for chemical dehydration for 20 min. All the cover slips were then dried for one day and sputter-coated with a thin gold film. The samples were viewed with scanning electron microscopy (SEM, Hitachi, S-3000 N) in high-vacuum mode at 20 kV.

### Outer membrane permeabilization activity

The permeability of the outer membrane of *E.coli* was verified by the NPN assays. The *E. coli* cells were cultured to the logarithmic phase, collected by centrifugation, washed with PBS twice, and re-suspended in a tube. The resuspended cells (10^8^ CFU/mL, 1 mL) were incubated with Ce6-TPP-PEI, TPP-PEI, or free Ce6 at 37 °C for 30 min. Subsequently, the cells were collected, washed with PBS to remove the residual particles, and irradiated or not irradiated with 630 nm LED light at a light intensity of 30 mW/cm^2^ for 5 min or not. Then 10 μL of NPN solution (2 mM, ethanol) was added to each sample, and fluorescence spectra (Ex = 350 nm, Em = 429 nm.) were recorded at a fixed time. The PBS group and NPN was set as the control group.

### Action of Ce6-TPP-PEI on *E. coli* cell structure (TEM)

*E. coli* was cultured to the logarithmic phase, collected by centrifugation, and washed with PBS twice, followed by concentration and re-suspension in a tube. Then the cells in each tube were incubated with Ce6-TPP-PEI, TPP-PEI, or free Ce6 at 37 °C for 30 min. Then, the cells were collected by centrifugation and washed with PBS to remove the residual particles, then re-suspended and irradiated with 630 nm LED light at a dose of 30 mW/cm^2^ for 5 min. Subsequently, the *E. coli* cells were collected and 2.5% glutaraldehyde was added to each tube to further prepare the cells for ultrathin sections. TEM (Hitachi, H-7650) was used to record the morphology of *E. coli*. PBS served as the control group.

### Hemolysis assay

A rabbit blood sample (4%) was taken and centrifuged at 1000 rpm for 5 min to harvest red blood cells (RBCs), which were washed with PBS (pH = 7.2, 0.01 M) three times until the supernatant was colorless. Then, 100 µL of different concentration gradients of Ce6-TPP-PEI was added to an EP tube. Subsequently, another 900 µL of 2% RBC suspension was added into the EP tube. After incubated at 37 °C for 2 h, the tube was centrifuged at 1000 rpm for 5 min to collect the supernatant for further testing. The absorbance of the supernatant was recorded at 541 nm by a microplate reader (BioTek, Synergy H1). PBS (900 µL, pH = 7.2, 0.01 M) and deionized water (100 µL) mixed with the RBCs suspension (800 µL), served as the negative control group and the positive control groups, respectively. The hemolysis rate was calculated according to the following formula:

$${\text{Hemolysis rate }}\left( \% \right) = {{{\text{A}}_{{{\text{sample}}}} - {\text{A}}_{{{\text{negative}}}} } \mathord{\left/ {\vphantom {{{\text{A}}_{{{\text{sample}}}} - {\text{A}}_{{{\text{negative}}}} } {{\text{A}}_{{{\text{positive}}}} - {\text{A}}_{{{\text{negative}}}} }}} \right. \kern-\nulldelimiterspace} {{\text{A}}_{{{\text{positive}}}} - {\text{A}}_{{{\text{negative}}}} }} \times 100$$

### Resistance assay

The changes in the MIC values of normal *E. coli* strains were estimated before and after incubation to determine resistance according to previous literature [40]. Briefly, the bacteria were cultured and pre-treated as described above in “Confocal laser scanning microscopy observation of cellular uptake”. Then, 100 µL of medium containing a serial dilution of Ce6 (0–1.870 μg/mL) and bacterial solution (10^5^ CFU/mL, 100 μL) were added to the well of a 96-well plate, respectively. After incubation for 24 h, the MIC value for planktonic cells was defined as the lowest concentration of antibiotics without visible bacterial growth. Subsequently, 20 generations of bacteria were tested at half the MIC of Ce6-TPP-PEI. The bacteria were finally collected to retest the MIC value.

### Statistical analysis

Statistical analyses were performed using Student’s t-test. P < 0.05 was considered significant. Data were expressed as mean ± SD.

## Results and discussion

### Preparation and characterization of the polymer

**Scheme 1:**
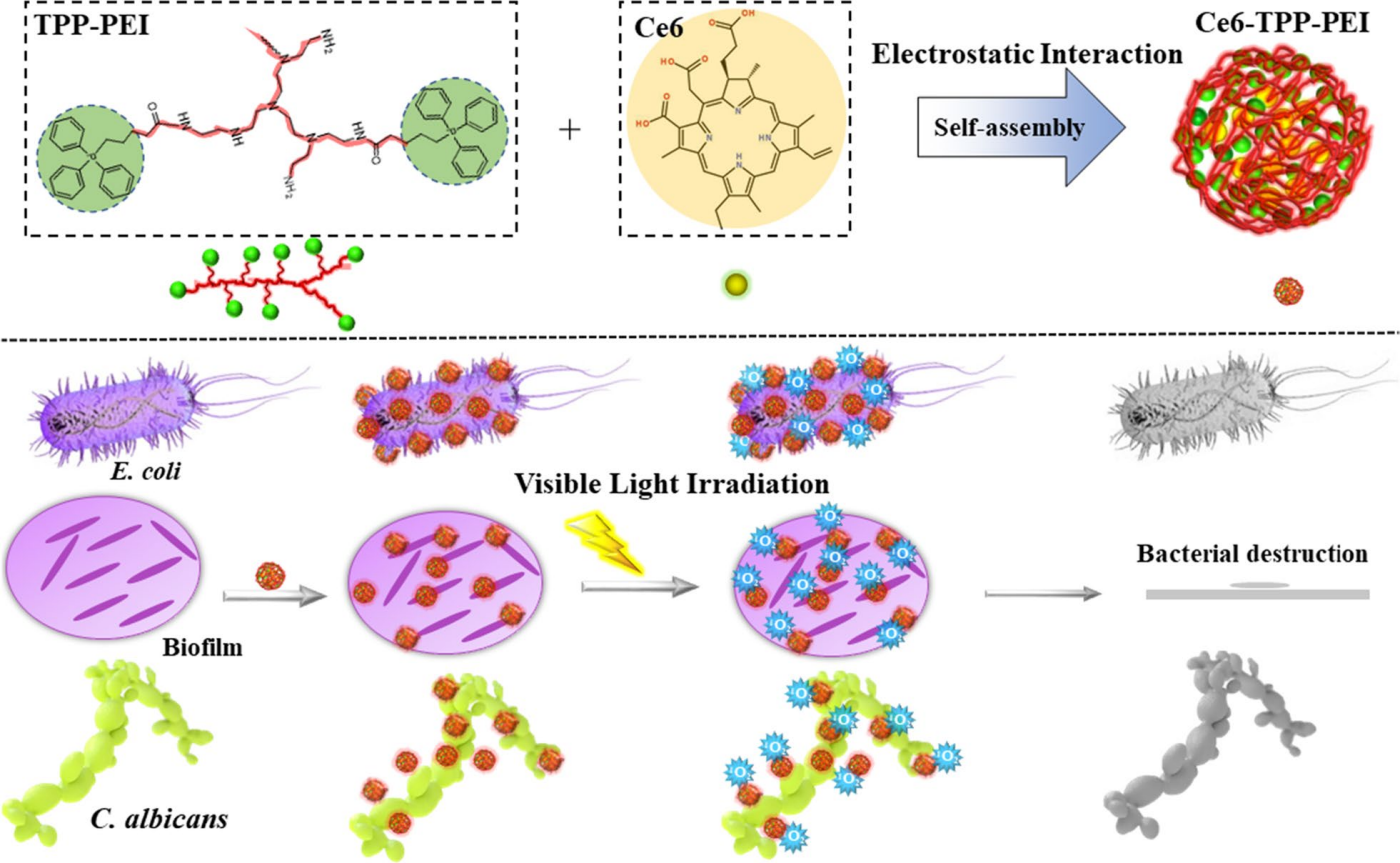
Schematic illustration of the fabrication process of the amphiphilic polymer (TPP-PEI) and photosensitizer loading for Ce6-TPP-PEI, as well as evaluation of anti-pathogen and biofilm removal upon light irradiation

The fabrication process of the amphiphilic polymer (TPP-PEI) and photosensitizer loading for Ce6-TPP-PEI, as well as the evaluation of the anti-pathogenic effect upon light irradiation, are illustrated in Scheme 1.

**Fig. 1.**
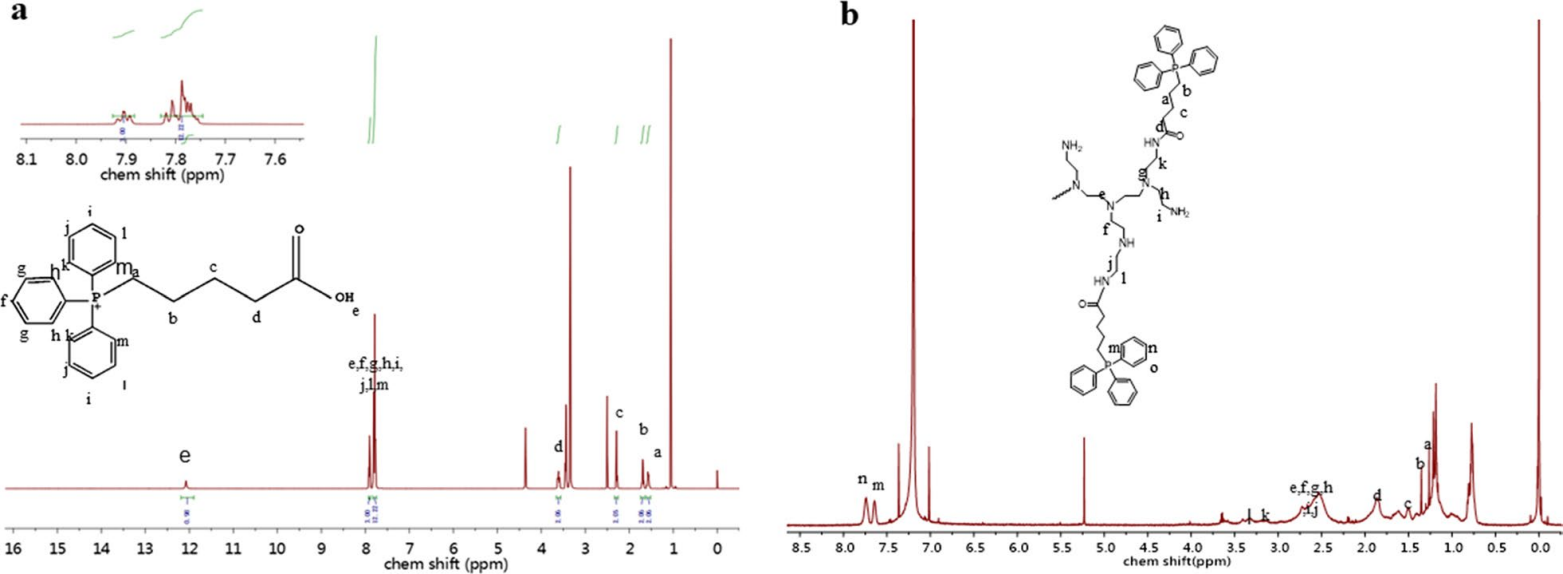
^1^HNMR spectra of TPP (**a**) and amphiphilic polymer TPP-PEI (**b**)

The polymer (TPP-PEI) was regulated by adjusting the hydrophobic ratio of the TPP, expecting to find the most appropriate molecular weight for the branched polymer which could self-assemble to form micellar aggregates in water and exhibit an antibacterial effect. A detailed synthetic methodology was described in the experimental “Preparation of TPP-PEI and Ce6-TPP-PEI” and Additional file 1: Fig. S1. To synthesize the branched polymer with TPP units in the polymer backbone, a 5-bromovaleric acid was reacted with the triphenylphosphine to obtain TPP, then the amphiphilic polymer TPP-PEI was fabricated from commercially available polyethylenimine and TPP by an amidation reaction. Their chemical structure was verified by ^1^HNMR (Fig. 1).

**Fig. 2:**
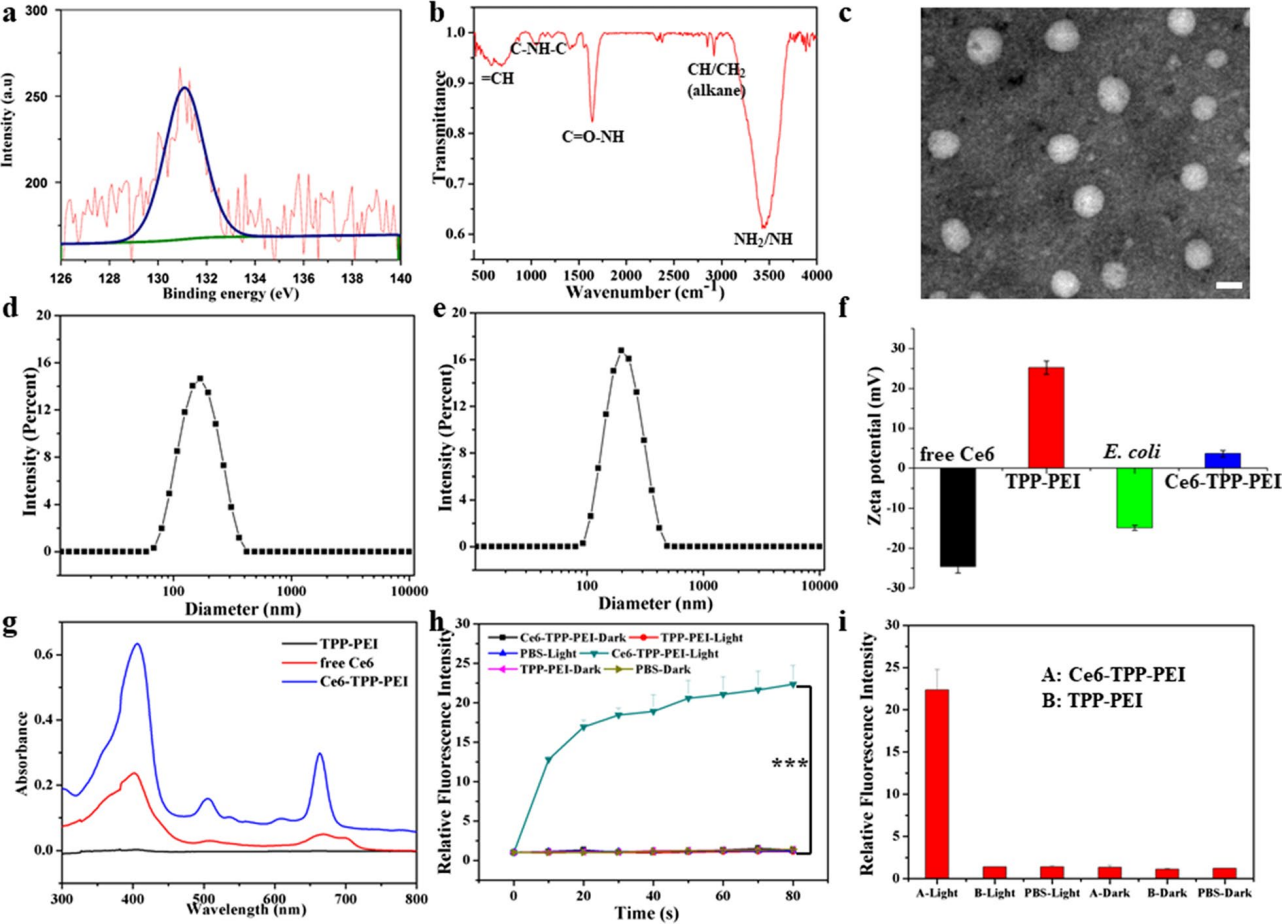
XPS spectra of TPP-PEI (**a**). FT-IR of TPP-PEI (**b**). TEM image of Ce6-TPP-PEI. The scale bar corresponds to 80 nm (**c**). Average size distribution TPP-PEI (**d**) and Ce6- TPP-PEI (**e**). The electrostatic surface potential of free Ce6, TPP-PEI, *E. coli* cell and Ce6-TPP-PEI in an 0.9% NaCl solution, the data are presented as mean ± SD (n = 3) (**f**). Absorption spectra of free Ce6, TPP-PEI, and Ce6-TPP-PEI in pure water (**g**). Relative fluorescence intensity of the ROS of a solution of Ce6-TPP-PEI or TPP-PEI in the presence or absence of light irradiation (30 mW/cm^2^). DCFH was used as a probe, and PBS was set as a control. The data are presented as mean ± SD (n = 3) (h). Fluorescent detection of ROS generation of a solution of Ce6-TPP-PEI or TPP-PEI in the presence or absence of light irradiation (30 mW/cm, 80 s). DCFH was used as a probe, and PBS was set as a control. The data are presented as mean ± SD (n = 3) (i)

From x-ray photoelectron spectra (XPS), the P_2p_ (130 eV) peaks were observed from TPP-PEI (Fig. 2a), indicating that TPP was successfully grafted to PEI. The characteristic peak at 1649 cm^−1^, assigned to C = O was also observed in the FT-IR spectra of TPP-PEI. Moreover, the existence of characteristics of the benzene ring (860–670 cm^−1^) also confirmed that TPP was coated onto the PEI (Fig. 2b). Gel permeation chromatography (GPC) was used to determine the molecular weights and molecular weight distribution of TPP-PEI. The Mw was 44 kDa with a photodynamic inactivation (PDI) of 2.59 (Additional file 1: Fig. S2). The rate of substitution degree of TPP in TPP-PEI was analyzed by element analysis (EA). By calculating the mass fraction of N, the molar ratio of repetitive units of PEI and TPP was found to be 8.3.

The Ce6-TPP-PEI micelles were prepared from the amphiphilic TPP-PEI through dialysis. The standard curve of the absorbance at 405 nm *vs*. the concentration of Ce6 in DMSO was obtained to calculate the LC and EE of Ce6 in TPP-PEI (Additional file 1: Fig. S3), which were found to be 7.727% and 85.29%. The average sizes and nanostructure of the particles were analyzed by TEM (Fig. 2c) and dynamic light scattering (DLS) (Fig. 2d, e), respectively. The average size of TPP-PEI measured from DLS analysis, was 134.2 nm, while the average size of Ce6-TPP-PEI was 142.6 nm. The slight increase in size as compared with Ce6 free micelles was attributed to the loading of photosensitizer Ce6. The electrostatic surface potential of Ce6-TPP-PEI and free Ce6 were determined using a Zeta-sizer Nano Series instrument (Fig. 2f). The zeta potential of TPP-PEI was 25.2 mV, while Ce6-TPP-PEI was found to be 3.63 mV in an 0.9% NaCl solution. The reduction of the electrostatic surface potential of Ce6-TPP-PEI would be a benefit for its biocompatibility. Meanwhile, the zeta potential value was -14.9 mV for *E. coli* under the same conditions. Most notably, the electrostatic interaction between positively charged polymers and the negatively charged cell wall is a vital superiority for enhanced light-activated anti-pathogen properties due to the possibility of efficient diffusion of ROS produced by PS into the cell membrane. The absorption spectra of free Ce6, TPP-PEI, and Ce6-TPP-PEI were recorded by spectrophotometry to further confirm the successful loading of free Ce6 to TPP-PEI (Fig. 2g).

### The detection of reactive oxygen species

The generation of ROS is one of the vital steps concerned with the photodynamic inactivation of pathogens. To verify the ability of Ce6-TPP-PEI to produce ROS, DCFH solution, from the hydrolysis of DCFH-DA, served as a fluorescent label to measure the ROS contents separately treated with Ce6-TPP-PEI and TPP-PEI. As shown in (Fig. 2h, i), with the same DCFH concentration in all samples, a weak fluorescence signal was detected in the mixture of DCFH and Ce6-TPP-PEI, or the mixture of DCFH and TPP-PEI, indicating that no ROS was generated when DCFH was mixed with Ce6-TPP-PEI or TPP-PEI without light irradiation. Along with light irradiation, DCFH alone or the mixture of DCFH and TPP-PEI still did not produce ROS. In sharp contrast, there was significant increment of fluorescence intensities at 525 nm in the case of Ce6-TPP-PEI and light irradiation, due to the generation of ROS from encapsulated Ce6 in the micelles irradiated with LED light. Meanwhile, with a fixed light irradiation intensity (30 mW/cm^2^), an almost linear relationship was observed between the fluorescence intensities of the generated DCF and the light irradiation time.

### Confocal imaging of bacterial labeling

**Fig. 3:**
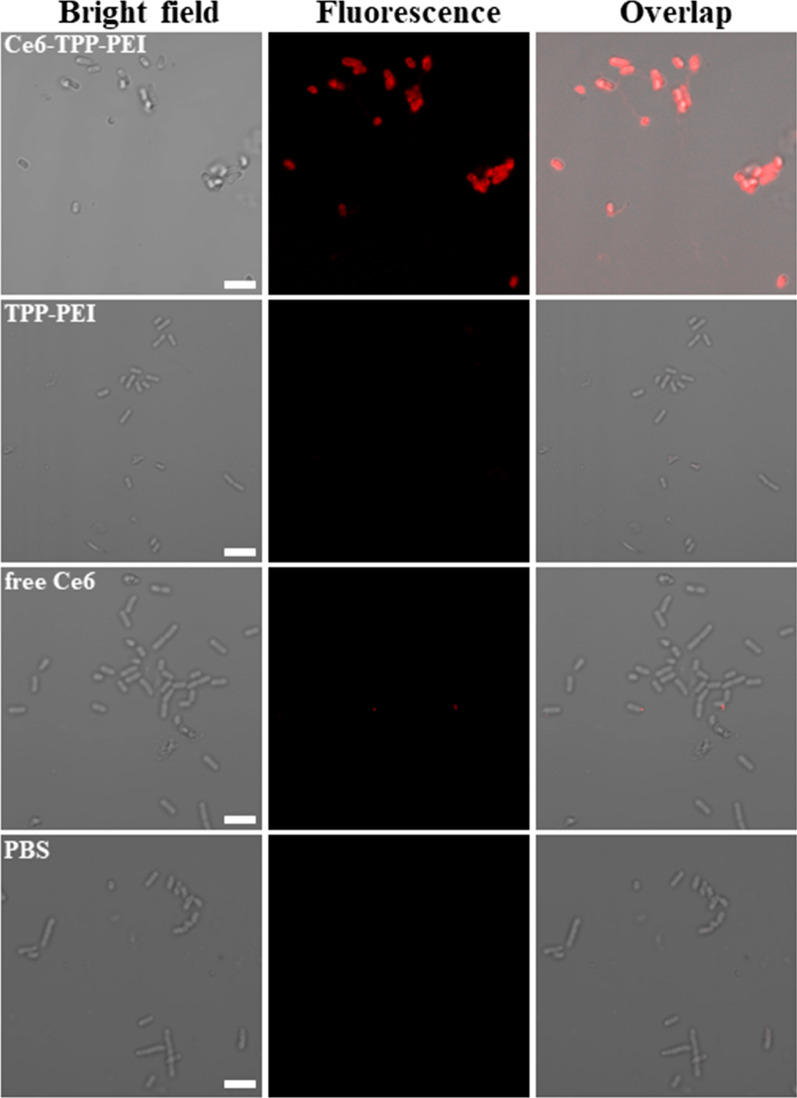
CLSM images of *E. coli* suspensions incubated with Ce6-TPP-PEI, TPP-PEI, or free Ce6 for 30 min. The scale bar is 3 μm

The efficiency of ROS is limited by the short ^1^O_2_ lifetime (< 4 μs) and limited diffusion distance (0.01–0.02 μm) [41]. Based on our design, the primary purpose of the TPP-PEI was to enhance the binding affinity of Ce6 for bacterial cell membranes. To confirm the targeting of Ce6-TPP-PEI to typical Gram-negative bacteria cells, the fluorescent images of *E. coli* cells incubated with Ce6-TPP-PEI were visualized by CLSM. As shown in Fig. 3, a red fluorescence signal was observed on the surface of *E. coli* cells, indicating the successful and precise positioning of Ce6-TPP-PEI on the surface of *E. coli*. Additionally, after treatment with free Ce6, almost negligible red fluorescence was observed in *E. coli* cells. Similarly, the same phenomenon was observed for the TPP-PEI group. We concluded that Ce6-TPP-PEI was able to successfully penetrate the outer membrane barrier of *E. coli* through the combination of electrostatic and hydrophobic interactions between the nanoparticles and the bacteria, which is a key step for PACT to exert its full antimicrobial activity. Meanwhile, PS could also be used to identify the location and extent, with no need for extra tracer molecules in this design.

### Antibacterial activity

**Fig. 4:**
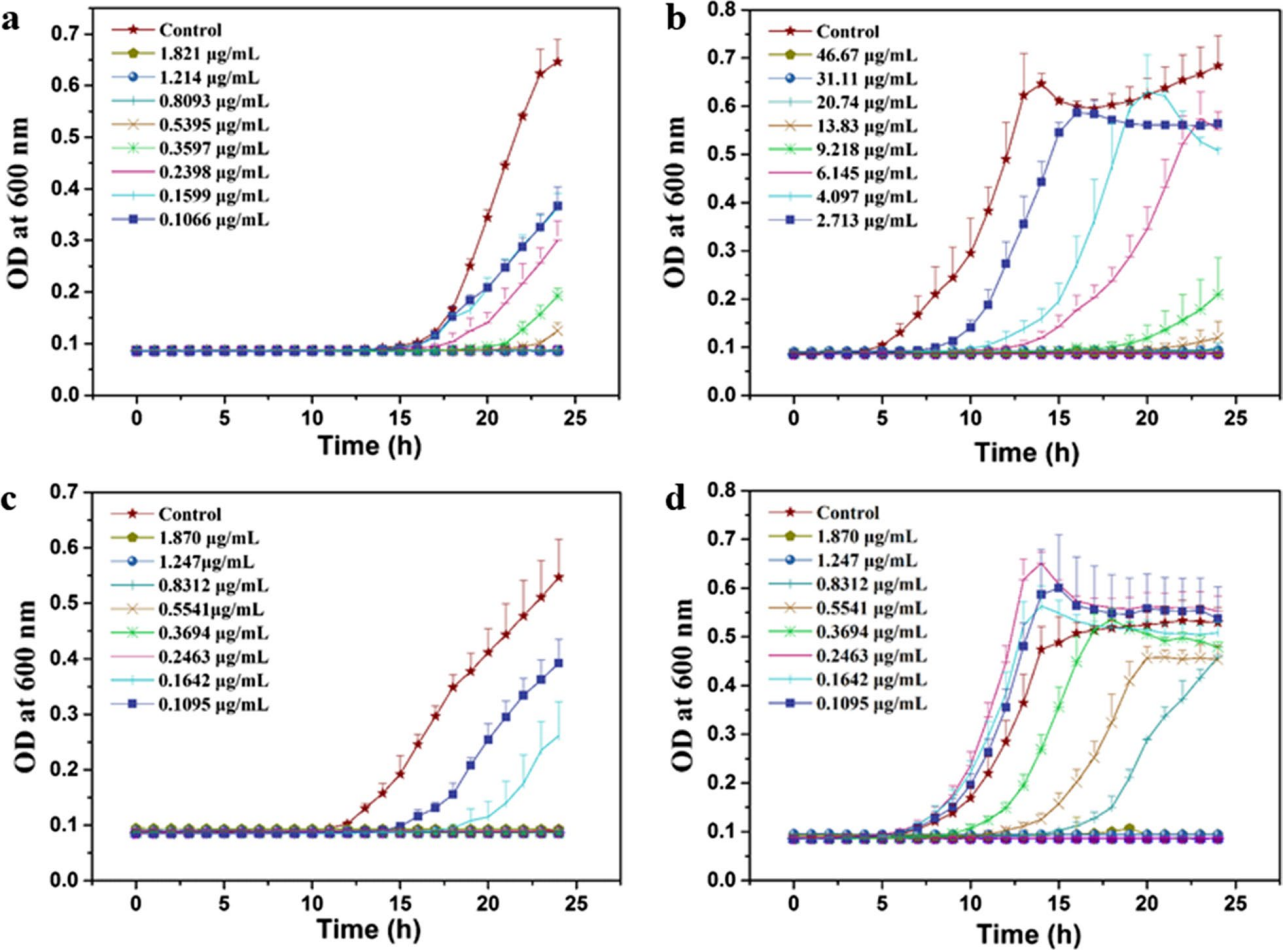
Antibacterial dynamic curves of TPP-PEI against *B. subtilis* (**a**) and *E. coli* (**b**) with irradiated. Antibacterial dynamic curves of Ce6-TPP-PEI against *B. subtilis* (**c**) and *E. coli* (**d**) with irradiation. Cells were grown in the presence of different concentrations of Ce6 or TPP-PEI for 24 h. The data are presented as mean ± SD (n = 4)

After confirming the excellent ability of Ce6-TPP-PEI to bind to the outer membrane of *E. coli* cells, we investigated whether it exhibited the desired antimicrobial activity under planktonic conditions. The kinetic curve of *E. coli* upon treatment with Ce6-TPP-PEI and TPP-PEI was performed to evaluate the efficiency of antibacterial activity. First, to assess the minimum inhibitory concentration (MIC), with fixed light irradiation intensity (30 mW/cm^2^) and time (5 min), the cell solutions (10^5^ cells/mL) were mixed with varying TPP concentrations (0.1066–46.67 μg/mL) and incubated with M9 media at 37 °C for 24 h to record OD_600_. Next, the MIC values of Ce6-TPP-PEI and TPP-PEI were evaluated by a turbidity-based assay. As shown in Fig. 4, three stages of bacterial growth were observed. In the initial 4 h was the lag phase, followed by the exponential phase and stabilization phases in the control group. Concerning the groups treated with Ce6-TPP-PEI or TPP-PEI, the growth of *E. coli* cells was completely inhibited during the first 8 h (4b, d). During the next 10 h, the cells at the lower dose of TPP-PEI began to enter the logarithmic phase gradually. Encouragingly, the higher dose of Ce6-TPP-PEI or TPP-PEI continued to completely inhibit the growth of bacteria for at least 24 h.

*Bacillus* species also cause a wide variety of infections, and they are significant pathogens in humans with increasing frequency [42]. Therefore, *B. subtilis* was chosen as a typical Gram-positive bacterial model to explore the antibacterial ability of Ce6-TPP-PEI and TPP-PEI (4a, c). As expected, an excellent antibacterial ability could also be observed on *B. subtilis*. The MIC value of Ce6-TPP-PEI and TPP-PEI against *E. coli* and *B. subtilis* was summarized in Additional file 1: Table S1 (Supporting Information). Dose-dependent growth inhibition of TPP-PEI and Ce6-TPP-PEI against *B. subtilis* and *E. coli* was summarized in Additional file 1: Fig. S4.

### Comparison of antibacterial activity

**Fig. 5:**
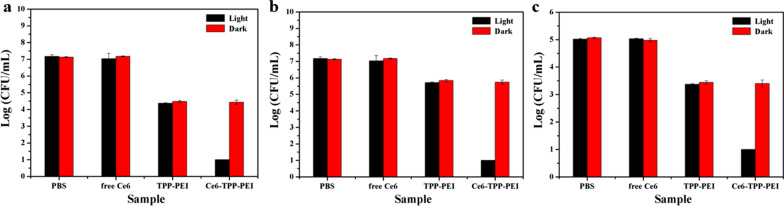
Cell density comparison of different samples against *E. coli* (**a**), *B. subtilis* (**b**) and *C. albicans* (**c**)

The plate counting method was carried out to further assess the antimicrobial efficacy of Ce6-TPP-PEI, TPP-PEI, and free Ce6 accurately. The logarithmic cell density was analyzed statistically according to the number of colonies counted after culturing with different samples. As shown in Fig. 5, irrespective of being in the dark or light, there was no apparent inhibitory effect on the free Ce6 and PBS groups. Meanwhile, there were no visible colonies in the Ce6-TPP-PEI group with light against *E. coli*, which is much lower than the 6 log10 CFU/mL in the PBS group. Ce6-TPP-PEI showed a 6-log inactivation of bacterial concentration (Fig. 5a, > 99.99% inactivation efficiency). A similar phenomenon was also observed in *B. subtilis* and *C. albicans*. *B. subtilis* could obtain 6-log inactivation of bacteria with the concentration of Ce6 was 0.48 μg/mL (Fig. 5b). According to the count colony forming units, when the dose at Ce6 was 56 μg/mL, there were no visible colonies in the Ce6-TPP-PEI group with light against *C. albicans*, which is lower than 4 log10 cfu/mL in the PBS group (Fig. 5c). It indicated that both Ce6-TPP-PEI showed a 4-log inactivation of bacterial concentration (> 99.99% inactivation efficiency). These results suggested two points. First, the combination of PACT and cationic polymer had a good effect. Second, TPP-PEI alone not only exhibited an antibacterial effect but also notably enhanced the antimicrobial activity of free Ce6, which was negligible which was negligible even the dose at Ce6 was 16.67 μg/mL.

### Live/Dead bacterial viability assay

The live/dead staining method was also chosen to estimate the excellent antimicrobial efficiency of the Ce6-TPP-PEI against a model bacteria of *E. coli*. SYTO 9 and PI were the fluorescent dyes for fluorescence imaging. According to the manufacturer’s instructions, *E. coli* was first separately pre-treated with Ce6-TPP-PEI, Ce6-TPP-PEI, and free Ce6, illuminated under an LED light (630 nm, 30 mW/cm^2^, 5 min), and then incubated with a mixture of SYTO9/PI for 20 min in the dark. The bacterial cells alone served as a control. CLSM and fluorescence spectroscopy were separately adopted to directly and indirectly detect red and green signals.

**Fig. 6:**
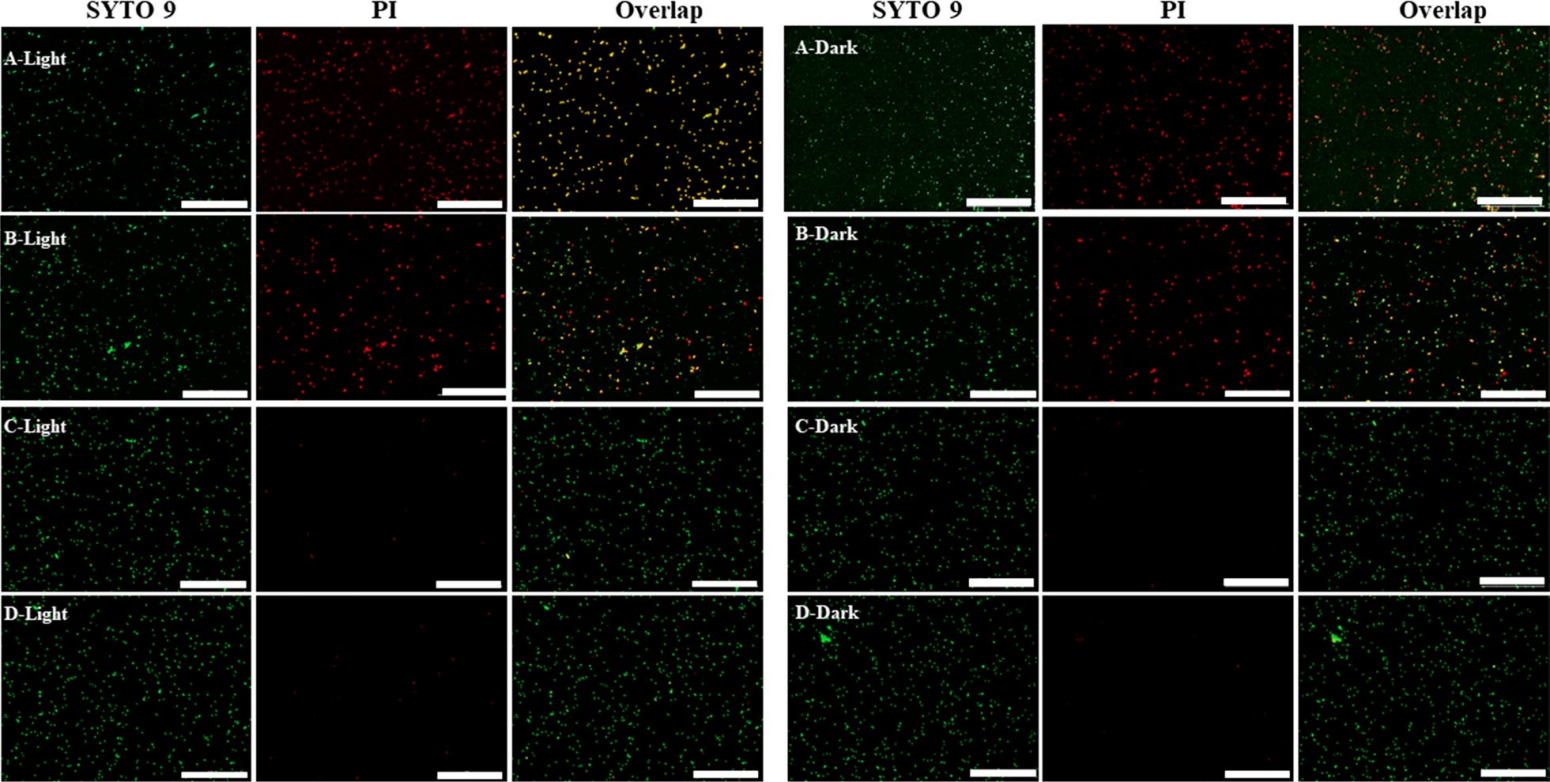
CLSM images for fluorescent detection of *E. coli* suspensions stained with SYTO/PI after being treated with Ce6-TPP-PEI (A), TPP-PEI (B), free Ce6 (C) and PBS (D). The concentration of Ce6 was 1.746 µg/mL (Scale bar: 30 µm)

As presented in Fig. 6, no matter in the dark or light, there was no apparent red fluorescence signal in the cells treated with free Ce6 and PBS, indicating no outer membrane damage of the cells. In addition, the inhibitory effect on the growth of bacteria was negligible. By contrast, in the absence of light irradiation, *E. coli* cells treated with TPP-PEI or Ce6-TPP-PEI could both be observed with similar green/red ratio fluorescence signals. Furthermore, a portion of cells could only be stained by SYTO 9, which might be attributed to the limited antibacterial activity of TPP-PEI. In the presence of light irradiation, relatively significant red fluorescence was observed in cells treated with Ce6-TPP-PEI, indicating enhanced sterilizing efficiency. However, after treatment with TPP-PEI, significantly weaker red fluorescence was observed in cells, similar to the intensity in cells treated with TPP-PEI or Ce6-TPP-PEI in the dark. These results suggested two points. First, given the same concentration of TPP-PEI, in the group of Ce6-TPP-PEI, the combination of PACT and cationic polymer exhibited an enhanced effect. Second, TPP-PEI alone also an exhibited antibacterial effect. These results were completely consistent with the antibacterial kinetic curve.

**Fig. 7:**
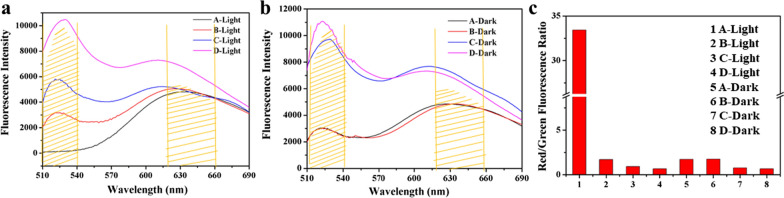
**a** Fluorescence spectra of *E. coli* suspensions stained with SYTO/PI after Ce6-TPP-PEI (A), TPP-PEI (B), free Ce6 (C) and PBS (D) in the presence (5 min at 30 mW/cm^2^) or absence of visible light irradiation (**b**). The red/green fluorescence ratios were calculated (**c**). The concentration of Ce6 was 1.746 µg/mL

As shown in Fig. 7, with light irradiation, the red fluorescence in the Ce6-TPP-PEI group was significantly enhanced compared with the TPP-PEI group. Furthermore, the red/green fluorescence ratios were calculated through the integrated fluorescence. Higher ratio values indicated superior antibacterial efficiency (Fig. 7c), clearly demonstrating that the Ce6-TPP-PEI group exhibited better antibacterial efficiency due to its higher value of red/green fluorescence ratio.

### Inhibition of biofilm formation and biofilm removal capabilities

**Fig. 8:**
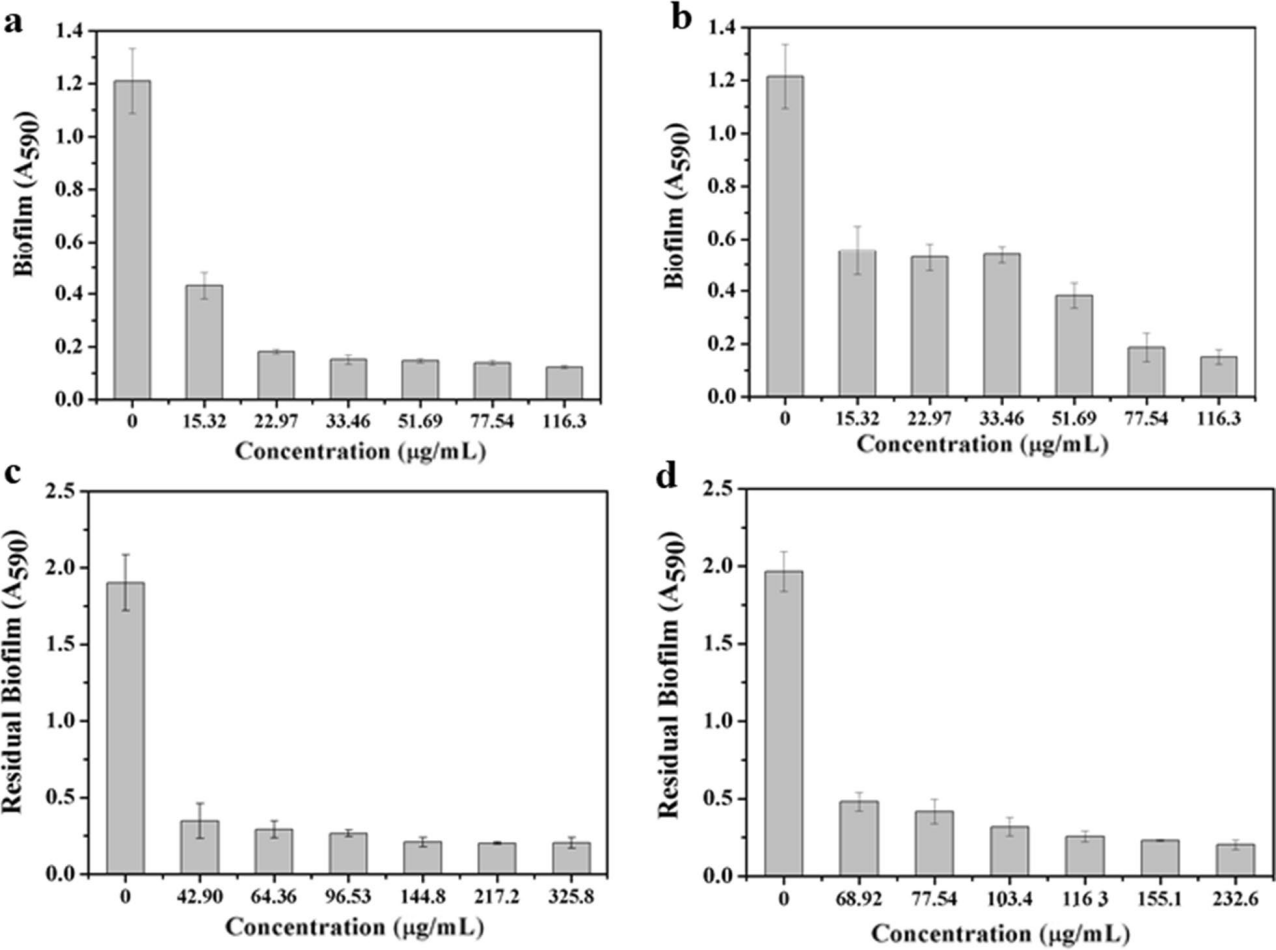
In vitro inhibition of *E. coli* biofilm formation by different concentrations of Ce6-TPP-PEI (**a**) and TPP-PEI (**b**) with LED light irradiation (5 min, 30 mW cm^−2^); 0.9% NaCl served as controls. In vitro biofilm removal capabilities on mature biofilm of Ce6-TPP-PEI (**c**) and TPP-PEI (d) with LED light irradiation (5 min, 30 mW cm^−2^)

Encouraged by the extraordinary synergistic antibacterial effect, we carried out a crystal violet (CV) staining assay to further explore the ability of the combination of PACT and cationic polymer to inhibit biofilm formation and ablation biofilms. As presented in Fig. 8a, all concentrations of TPP-PEI and Ce6-TPP-PEI nanoparticles exhibited varied inhibitory effects on biofilm formation. In the presence of light, with the same initial concentration of planktonic cells (10^8^ CFU/mL), at the TPP-PEI concentration of 22.97 μg/mL, Ce6-TPP-PEI caused a significant decrease in biofilm production, while TPP-PEI showed a relatively slight effect on inhibition of biofilm formation at the same concentration. Overall, a higher concentration of TPP-PEI alone was necessary to completely inhibit the formation of *E. coli* biofilms, which was attributed to the lack of ROS generation. Range from the entire tested concentration, it should be emphasized is that the inhibitory effect of Ce6-TPP-PEI was always higher than the TPP-PEI when the concentration of TPP-PEI was consistent.

Apart from exploring the ability of Ce6-TPP-PEI to inhibit biofilm formation, we also further explored the ability of Ce6-TPP-PEI to remove the formed biofilms. *E. coli* (10^8^ CFU/mL) was firstly pre-seeded to a 48-well plate for 48 h to form biofilms. Then the formed biofilms were treated with Ce6-TPP-PEI or TPP-PEI. Figure 8 shows the CV staining results for the residual biofilms after treatment with varying concentrations of Ce6-TPP-PEI or TPP-PEI and light irradiation. In the control group, *E. coli* performed robust biofilms on the solid–liquid interface. In the other group treated with Ce6-TPP-PEI or TPP-PEI, varying levels of residual biofilm could be observed. Up to test concentrations of 42.90 μg/mL of Ce6-TPP-PEI, it could even be found that the formed biofilm was completely removed. In contrast, the formed biofilm could not be completely removed even up to a concentration of 68.92 μg/mL of TPP-PEI. Meanwhile, the removal capacity depended on the tested concentration of TPP-PEI. Together, these results clearly demonstrated that with the same initial concentration of TPP-PEI, limited eradication effects were observed after treatment with TPP-PEI. In sharp contrast, in the biofilms treated with Ce6-TPP-PEI and light, the eradication effect was significantly enhanced, ascribed to the efficient antibacterial effect mediated by the PDT and cation polymer.

### Visualize the viability of the bacteria in the residual biofilm.

**Fig. 9:**
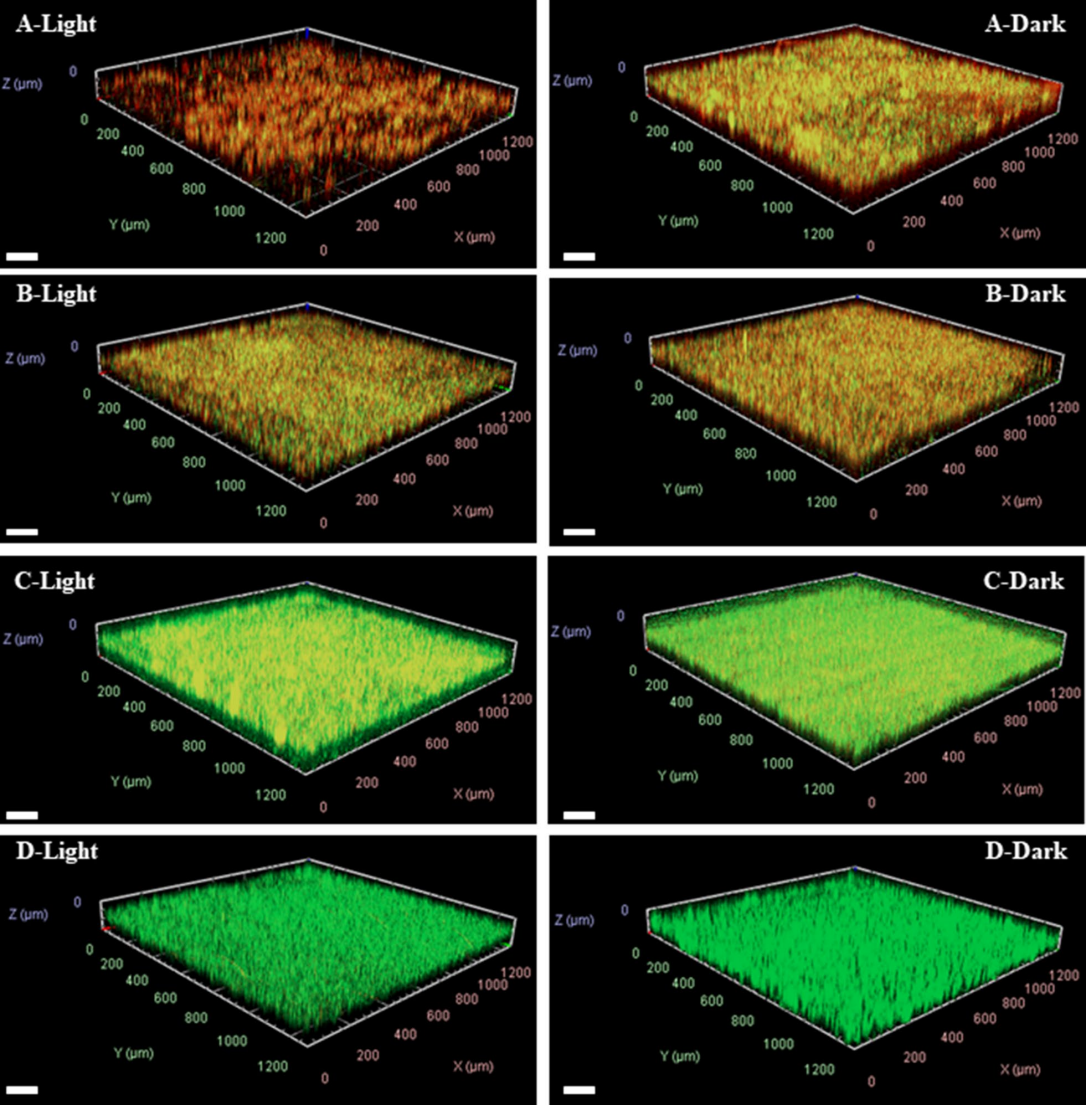
CLSM images of the *E. coli* biofilm eradication effect after treatment with Ce6-TPP-PEI (A), TPP-PEI (B), free Ce6 (C), and PBS (D) upon irradiating (5 min at 30 mW/cm^2^) or not. The biofilms were double-stained with SYTO 9 and PI. The concentration of Ce6 was 3.063 µg/mL (Scale bar: 200 μm)

A live/dead staining assay was also applied to further visualize the viability of the bacteria in the residual biofilm. Firstly, the *E. coli* biofilms were treated with Ce6-TPP-PEI, then was double-stained with SYTO 9 and PI, and finally observed by CLSM. As presented in Fig. 9, no matter illuminating or not illuminating, following treatment with free Ce6 or PBS, the biofilms exhibited high preservation of their three-dimensional (3D) architecture and extracellular matrices. This phenomenon of free Ce6 significantly contributed to the lack of targeting *E. coli* biofilms, and only a little PS could interact with bacteria after washing. However, with the same initial concentration of TPP-PEI, following treatment with Ce6-TPP-PEI and TPP-PEI, a strong red fluorescence signal appeared in the biofilm matrix, indicating a large number of dead bacterial. It is worth noting that even without light treatment, bacteria biofilms collapsed and were damaged to a certain extent after incubation with Ce6-TPP-PEI, suggesting it could enhanced the targeting of PS and ablate the formed biofilms. Furthermore, it is obvious that tin he biofilm treated with Ce6-TPP-PEI and light, the three-dimensional (3D) structure and extracellular matrix significantly collapsed, destroying their structural integrity, clearly indicating that the biofilms were wiped out in large quantities. These results showed the excellent ability of Ce6-TPP-PEI to eradicate existing biofilms, contributing to the excellent antibacterial effects of PDT and cation polymers. Meanwhile, Additional file 1: Figure S5 presents the depth of biofilms with different treatments.

### Antifungal activity

**Fig. 10:**
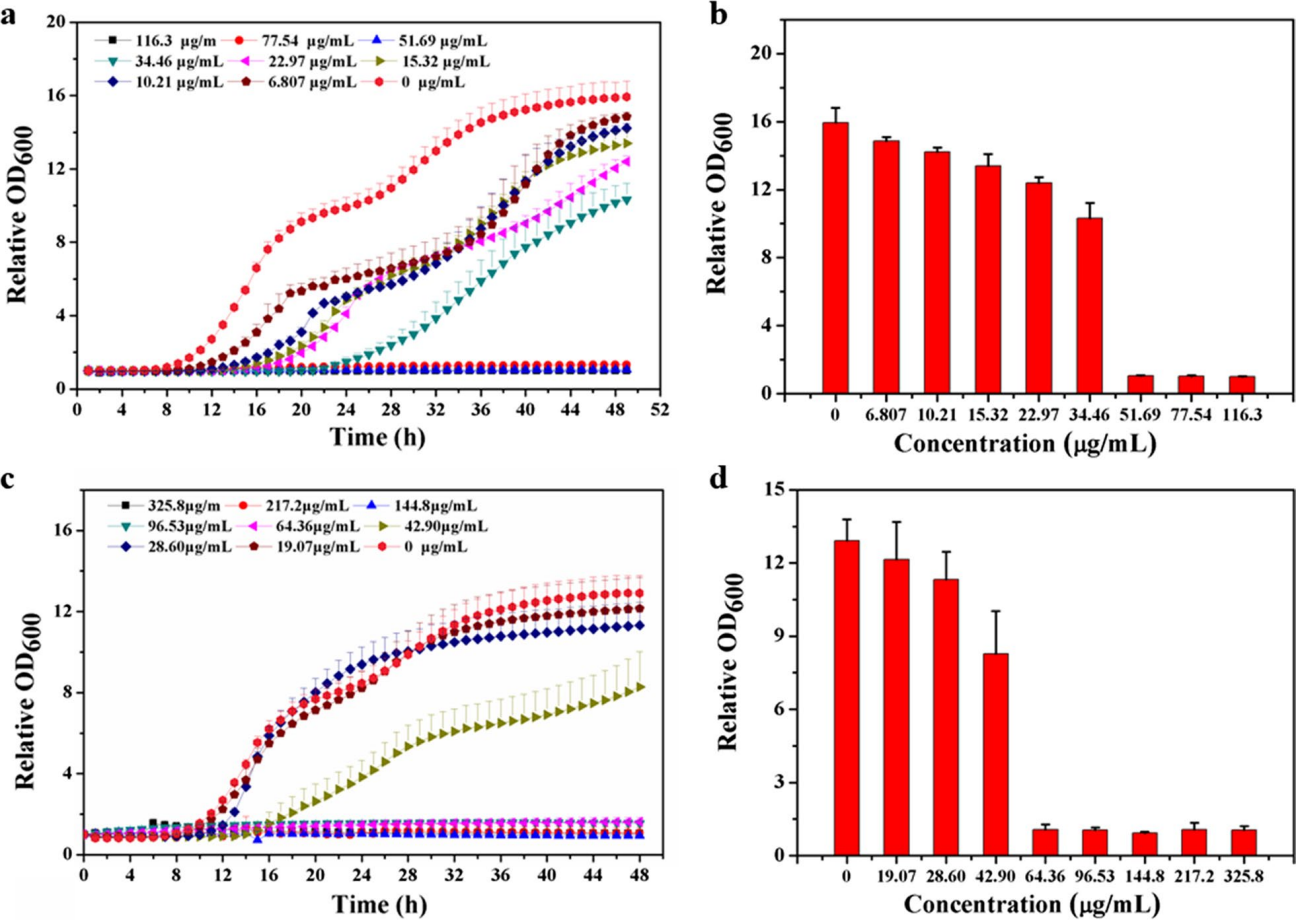
Inhibition of *C. albicans* planktonic cell growth under 5 min of light (λ = 630 nm, power = 30 mW/cm^2^) exposure with different concentrations of Ce6-TPP-PEI (**a**, **b**) or TPP-PEI (**c**, **d**). Each data point represents the mean and standard deviation of three replicates

*Candida* is one of the most common fungal pathogens, and *C. albicans* infection has the highest incidence rate among *Candida* species [43, 44]. Therefore, the antifungal activity of the Ce6-TPP-PEI against *C. albicans* under planktonic conditions was also fully explored. *C. albican* cells (10^4^, 100 μL) were mixed with varying concentrations of Ce6-TPP-PEI or TPP-PEI for 30 min and irradiated with visible light (630 nm, 5 min at 30 mW/cm^2^). As presented in Fig. 10, both Ce6-TPP-PEI and TPP-PEI exhibited antifungal activity. The lower activity of this concentration exhibited its relatively weak light inactivation. However, increasing the concentration improved the antifungal activity.

### The morphologie analysis of bacterial damage

**Fig. 11:**
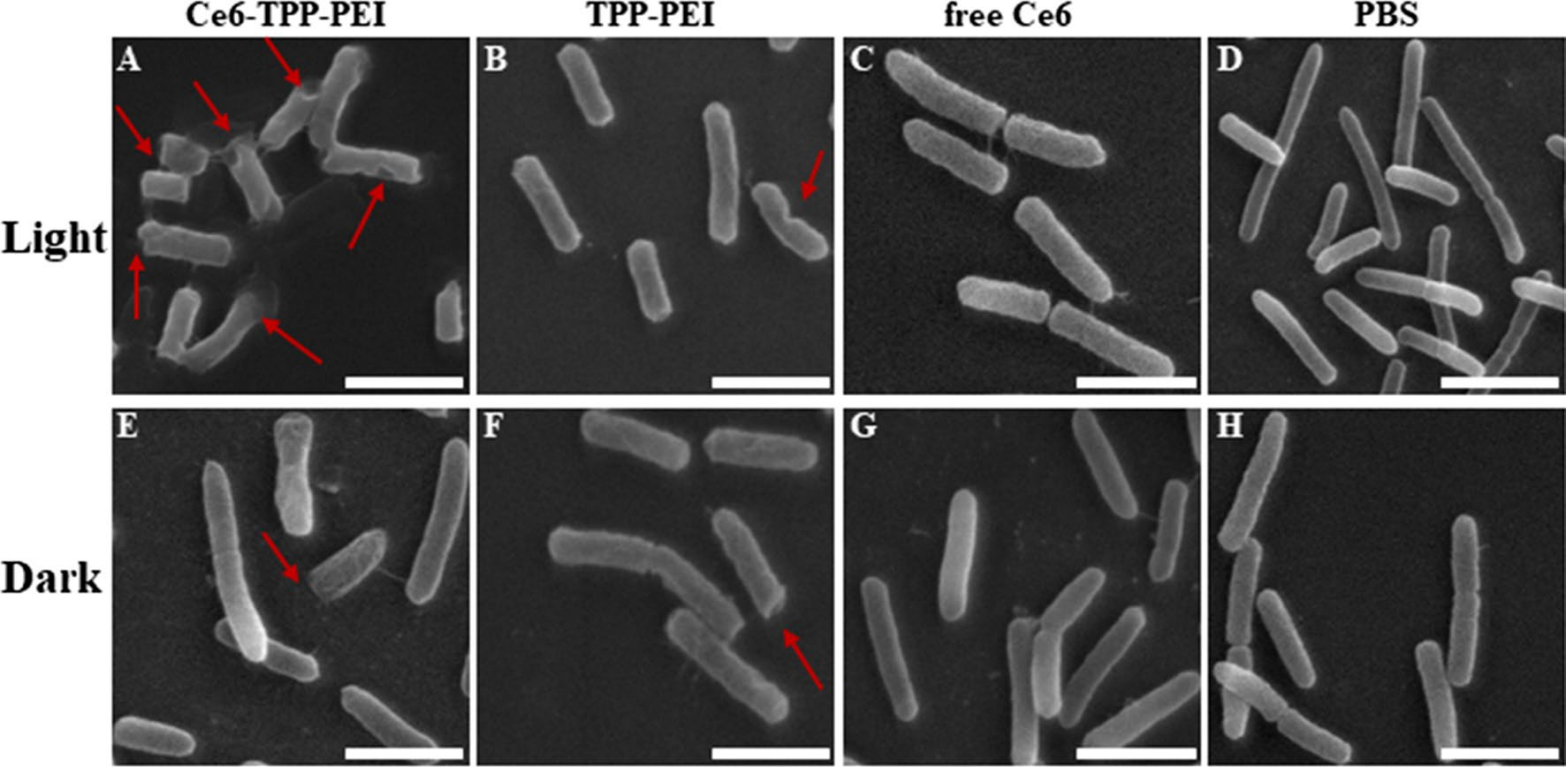
Scanning electron microscopy images of *E. coli* cells incubated with Ce6-TPP-PEI (**A**, **E**), TPP-PEI (**B**, **F**), free Ce6 (**C**, **G**) and PBS (**D**, **H**) with the same Ce6 or TPP-PEI concentration for 4 h with or without light (Scale bar: 3 μm). The concentration of Ce6 was 12 µg/mL. Arrows indicate *E. coli* integrity, where the cell membranes are destroyed and the cytoplasm flows out

To gain insights into the morphologies of *E. coli* after treatments with Ce6-TPP-PEI, followed by treatment with Ce6-TPP-PEI and a series of washes with phosphate buffer and organic solvents, SEM was performed to observe the planktonic bacteria. As shown in Fig. 11, hether with or without illumination, treatment with free Ce6 or PBS, no obvious damage was observed on the surface of the bacteria, and the cell wall exhibited intact morphology. In sharp contrast, in the absence of light irradiation, in *E. coli* with Ce6-TPP-PEI and TPP-PEI treatment, sporadic shrinkage could be observed on the surface of bacteria. As for in the presence of light radiation, there was no significant change in the degree of bacterial damage in the TPP-PEI group. However, a more flattened and damaged cell wall was observed in the Ce6-TPP-PEI group due to the enhanced PACT effect. This conclusion of SEM observations was also verified by the results of the CLSM observation of cellular uptake and antibacterial kinetics.

### Antibacterial mechanism

**Fig. 12:**
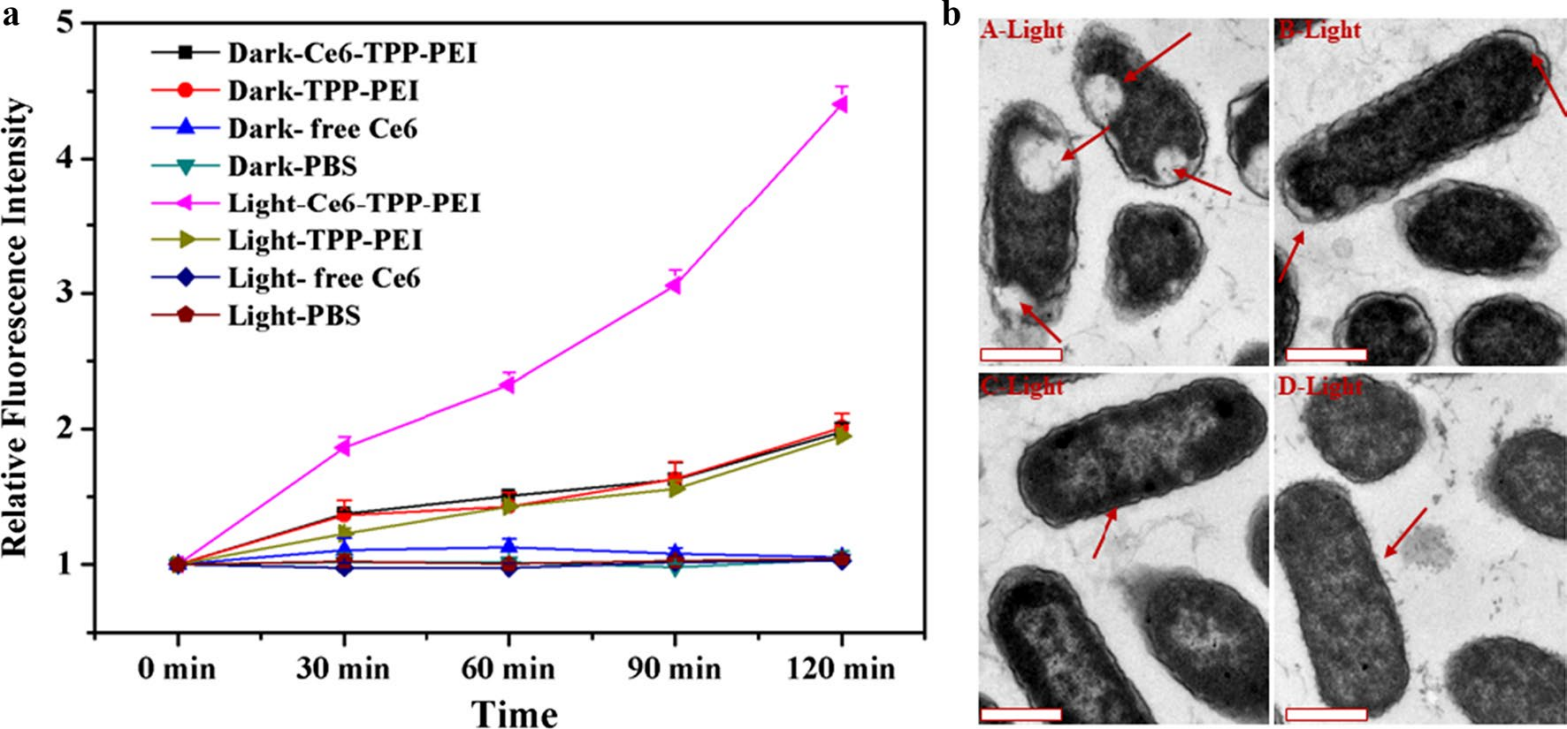
**a** The relative fluorescence emission intensity of NPN for *E. coli* suspensions treated with Ce6-TPP-PEI, TPP-PEI and free Ce6 light radiation or not. PBS was set as the control group. Data are presented as mean ± SD (n = 3). **b** TEM images of *E. coli* cells after being treated with Ce6-TPP-PEI, TPP-PEI and free Ce6 light radiation, Ce6-TPP-PEI (A), TPP-PEI (B), free Ce6 (C) and PBS (D), PBS was set as the control group. The concentration of Ce6 was 12 µg/mL (Scale bar: 500 nm). Arrows indicate the outer membrane and cytoplasmic membrane of *E. coli*

According to previous reports [42], the fluorescence intensity of NPN could indirectly reflect the integrity of *E. coli* outer membrane (OM). NPN emits weak fluorescence in a hydrophilic environment, but emits sharply strong fluorescence when entering a hydrophobic environment such as the OM of *E. coli*. Therefore, changes in the fluorescence intensity of NPN could indirectly reflect the integrity of the outer membrane. As shown in Fig. 12a, on matter with or without light radiation, there was a negligible change of NPN fluorescence intensity of free Ce6 and the control group, indicating that the cell structure was relatively intact. However, with the same initial concentration of TPP-PEI, the cells treated with Ce6-TPP-PEI and TPP-PEI showed an obvious fluorescence signal. Even without light treatment, there was a certain intensity of the fluorescent signal of NPN after incubation with Ce6-TPP-PEI, suggesting it could enhance the targeting of PS and destroy the outer membrane. Furthermore, it was evident that the cells treated with Ce6-TPP-PEI and light exhibited a strong fluorescent signal, indicating they had lost their structural integrity.

Cellular OM plays a crucial role in protecting cell viability and maintaining its morphology. Therefore, TEM was adopted to observe the changes in the OM of *E. coli* cells. As shown in Fig. 12b, in the presence of light irradiation, the OM and cytoplasm membrane of the PBS group remained smooth, and in the cells treated with free Ce6, the smooth OM exhibited insignificant ruffling. Meanwhile, cells treated with TPP-PEI, OM and cytoplasmic membrane (CM) were clearly separate, and the cytoplasm membrane was shrunken. Of particular note were the cells treated with Ce6-TPP-PEI, a pronounced separation of OM and CM was detected in these cells compared to cells treated with TPP-PEI, indicating that the integrity of *E. coli* was lost to a large extent, the cell membrane was severely disrupted, and the cytoplasm was effluxed. Considering all the results, such enhanced irreversible physical disruptions on *E. coli* cells were due to the induction of TPP-PEI, clearly suggesting the excellent ability of Ce6-TPP-PEI to eradicate bacteria, accounting for the notable antibacterial effects of PDT and cation polymers.

### Hemolysis assay

**Fig. 13:**
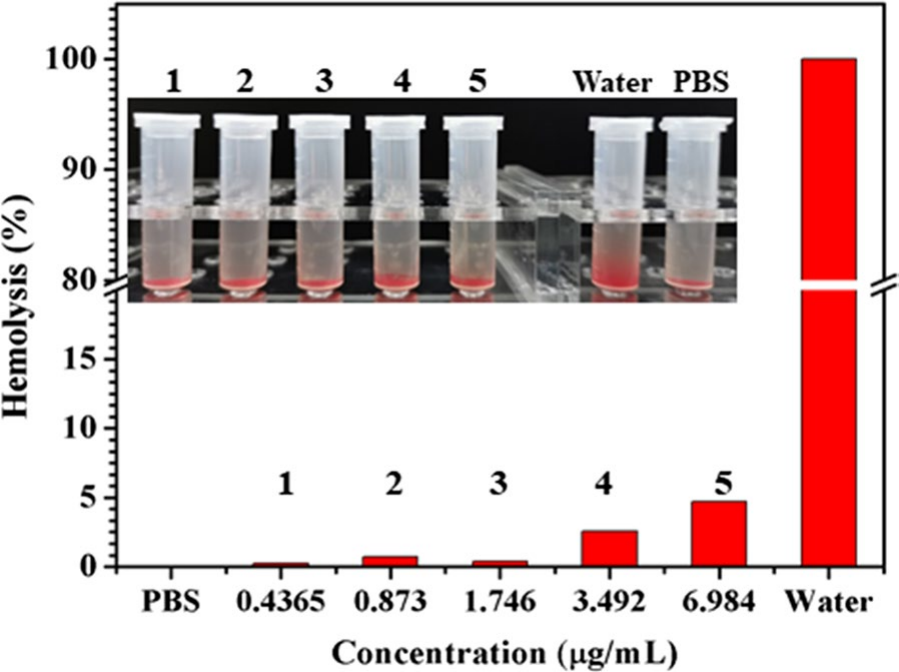
Hemolysis rates of Ce6-TPP-PEI with varied concentrations of Ce6, PBS and deionized water were set as negative and positive controls, respectively. Inset photographs: hemolysis assay of RBCs incubated with the micelles, compared to the positive (deionized water) and negative controls (PBS pH = 7.4)

A hemolysis assay was performed to determine the blood compatibility of Ce6-TPP-PEI. As shown in Fig. 13, in the concentration range of 0.4365–1.746 µg/mL of Ce6, the hemolysis rate of Ce6-TPP-PEI is considerably low (all < 5%). Meanwhile, the supernatants of the corresponding samples were colorless and transparent, with many red blood cells deposited on the bottom of the EP tube, indicating that the nanoparticles did not destroy the red blood cells. When the concentration was up to 3.492 µg/mL, which is much higher than MIC, the hemolysis rate was about 4%, which still did not exceed the national standard (5%) [45]. Therefore, it could be concluded that Ce6-TPP-PEI exhibited negligible obvious hemolysis, meeting the requirements of the hemolysis experiment of biomedical materials. The supernatant of the positive control group was obviously red, indicating that the red blood cells had been destroyed by the secondary water and hemolysis had occurred. Due to the favorable blood compatibility, these nanoparticles guaranteed the safety and effectiveness of their applications in the future.

### Resistance assay

Compared with the key-hole principle of antibiotics, there was no need for specific ligand (photosensitizer/polymer)–receptor interaction at or inside bacteria for light-based treatments [46, 47], suggesting that Ce6-TPP-PEI could not easily to develop drug resistance. To further understand the drug resistance of Ce6-TPP-PEI, the MIC value of Ce6-TPP-PEI against *E. coli* was estimated before and after incubation with the sample continuously to reflect resistance. When the concentration of Ce6-TPP-PEI reached 1.274 μg/mL with the broth microdilution method, the medium was still in a clear state visually after being cultured for 24 h, indicating that the MIC value was 1.274 μg/mL at the moment. After the bacteria multiplied for 20 generations under the continuous stimulation of Ce6-TPP-PEI with light irradiation (630 nm, 30 mW/cm^2^, 5 min), the MIC value against this bacterium was 1.911 μg/mL. The change in MIC value was less than 8 times, indicating that there was almost no drug resistance according to the Clinical and Laboratory Standards Institute (CISI).

## Conclusions

In summary, a novel visible light-induced nanoparticles platform with the complementary merits of the photodynamic and cationic polymer was developed for highly efficient anti-pathogenic agent and the ablation of biofilms. This system was constructed through an amphiphilic polyethyleneimine-based polymer with simple chemical conjugation from commercially available reagents. Then it could self-assemble into stable nanoparticles to provide hydrophobic cores for the encapsulation of Ce6 in an aqueous solution. Due to the electrostatic interaction, Ce6-TPP-PEI which enhanced targeting of PS could quickly enter the pathogen. Subsequently, when the light was on, the instantaneously generated ROS could inactivate most pathogens; then, the nanoparticles shell could prevent pathogen re-growth when the light was turned off. Thus, the combination of photodynamic therapy and cationic polymer exerted significant anti-pathogen and biofilm ablation effects. Finally, hemolysis assay demonstrated that Ce6-TPP-PEI exhibited good biocompatibility. Together, these findings suggested that Ce6-TPP-PEI is an ultra-efficient, anti-drug resistance, and biocompatible multifunctional antibacterial system, with application prospects as an anti-pathogenic agent, including bacteria and fungi, and for ablating biofilms.

## Supplementary Information

**Additional File 1**: **Figure S1**. Synthesis scheme of the TPP (a) and TPP-PEI polymer (b). **Figure S2**. Gel permeation chromatography of amphiphilic polymer TPP-PEI. **Figure S3**. UV/Vis spectra of Ce6-TPP-PEI with different amounts of Ce6 (a). Standard curve of free Ce6 by measuring absorbance at 405 nm with series of concentrations with UV/Vis spectra (b). **Figure. S4**. Dose-dependent growth inhibition of TPP-PEI against *B*. *subtilis* (a) and *E*. *coli* (b), Ce6-TPP-PEI against *B*. *subtilis* (c) and *E*. *coli* (d). Data are presented as mean ± SD (n=4). **Table S1**. Minimum inhibitory concentration (MIC) of Ce6-TPP-PEI, TPP-PEI, and free Ce6 against *B*. *subtilis*, *E*.*coli* and *C*. *albicans* (μg/mL). **Figure S5**. Composite 3-D micrographs of biofilms after treatment with Ce6-TPP-PEI (A), TPP-PEI (B), free Ce6 (C) and PBS (D) upon with visible light irradiation (5 min at 30 mW/cm^2^) or not. The biofilms were double-stained with SYTO 9 and PI.

## Data Availability

All data generated or analyzed during the current study are included in this published article and its Additional file 1.
