# Supplementary material for: Visible light-regulated cationic polymer coupled with photodynamic inactivation as an effective tool for pathogen and biofilm elimination
Source: J Nanobiotechnology. 2022 Nov 24;20:492. doi: 10.1186/s12951-022-01702-4 (PMC9694849; doi:10.1186/s12951-022-01702-4)
Supplement: Supplementary file 1 — Additional File 1: Figure S1. Synthesis scheme of the TPP (a) and TPP-PEI polymer (b). Figure S2. Gel permeation chromatography of amphiphilic polymer TPP-PEI. Figure S3. UV/Vis spectra of Ce6-TPP-PEI with different amounts of Ce6 (a). Standard curve of free Ce6 by measuring absorbance at 405 nm with series of concentrations with UV/Vis spectra (b). Figure. S4. Dose-dependent growth inhibition of TPP-PEI against B. subtilis (a) and E. coli (b), Ce6-TPP-PEI against B. subtilis (c) and E. coli (d). Data are presented as mean ± SD (n=4). Table S1. Minimum inhibitory concentration (MIC) of Ce6-TPP-PEI, TPP-PEI, and free Ce6 against B. subtilis, E.coli and C. albicans (μg/mL). Figure S5. Composite 3-D micrographs of biofilms after treatment with Ce6-TPP-PEI (A), TPP-PEI (B), free Ce6 (C) and PBS (D) upon with visible light irradiation (5 min at 30 mW/cm2) or not. The biofilms were double-stained with SYTO 9 and PI. [file 12951_2022_1702_MOESM1_ESM.docx]

**Visible light-regulated cationic polymer coupled with photodynamic inactivation as an effective tool for pathogen and biofilm elimination**

Qian Wang, Qingshan Shi, Yulian Li, Shunying Lu, Xiaobao Xie*

Guangdong Provincial Key Laboratory of Microbial Culture Collection and Application, State Key Laboratory of Applied Microbiology Southern China, Institute of Microbiology, Guangdong Academy of Sciences, Guangzhou 510070, P. R. China

*Corresponding author.

E-mail addresses: xiexb@gdim.cn (X. Xie)


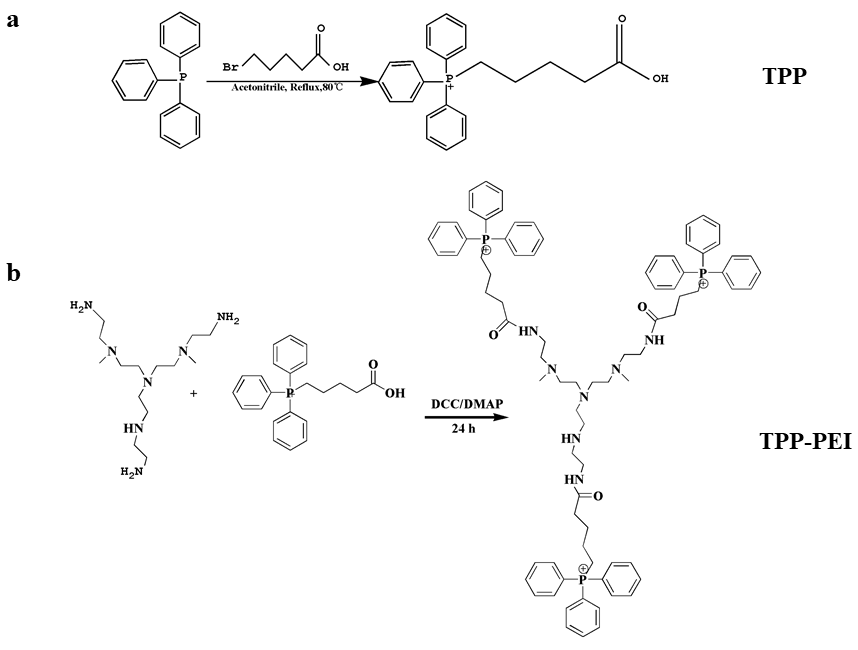


**Figure S1.** Synthesis scheme of the TPP (a) and TPP-PEI polymer (b).





**Figure S2.** Gel permeation chromatography of amphiphilic polymer TPP-PEI.


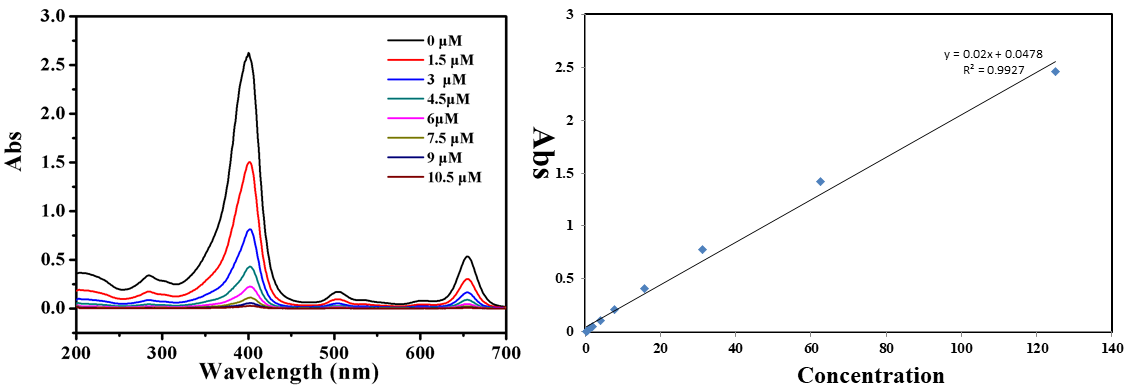


**Figure S3.** UV/Vis spectra of Ce6-TPP-PEI with different amounts of Ce6 (a). Standard curve of free Ce6 by measuring absorbance at 405 nm with series of concentrations with UV/vbvc Vis spectra (b).

**
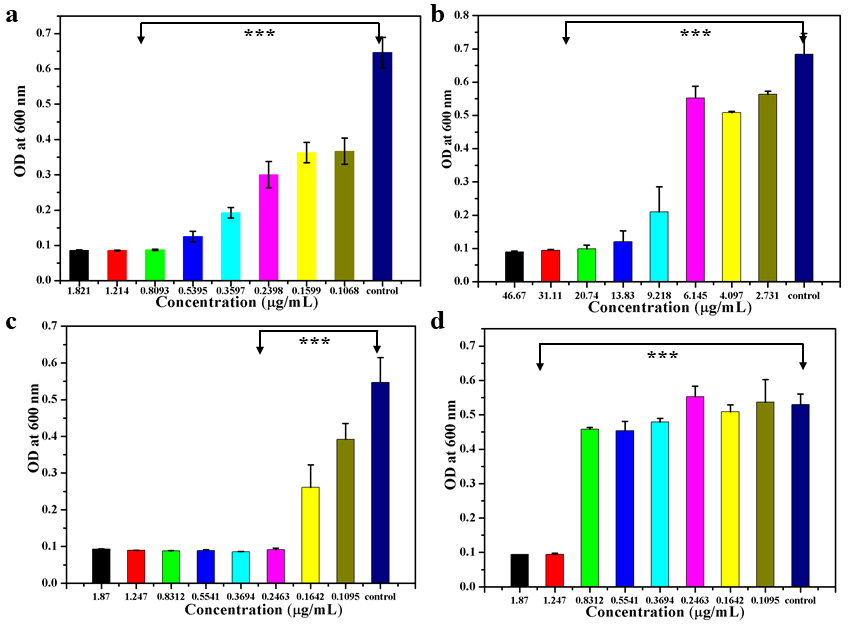
**

**Fig. S4.** Dose-dependent growth inhibition of TPP-PEI against *B. subtilis* (a) and *E. coli* (b), Ce6-TPP-PEI against *B. subtilis* (c) and *E. coli* (d). Data are presented as mean ± SD (n=4).

**Table S1** Minimum inhibitory concentration (MIC) of Ce6-TPP-PEI, TPP-PEI, and free Ce6 against *B.Subtilis*, *E.coli* and C. albicans (μg/mL).

| **Sample** | *B. Subtilis* | *E.coli* | *C. albicans* |
| --- | --- | --- | --- |
| TPP-PEI | 0.8093 | 20.74 | 64.36 |
| Ce6-TPP-PEI | 0.2463 | 1.274 | 51.69 |
| free Ce6 | > 3.292 | >16.67 | > 150 |


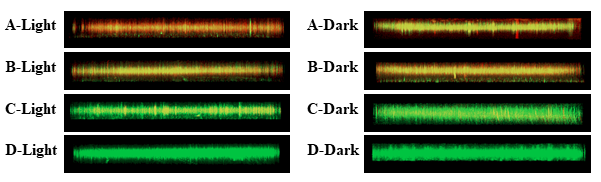


**Figure S5.** Composite 3-D micrographs of biofilms after treatment with Ce6-TPP-PEI (A), TPP-PEI (B), free Ce6 (C) and PBS (D) upon with visible light irradiation (5 min at 30 mW/cm^2^) or not. The biofilms were double-stained with SYTO 9 and PI.
